# Proteolytically Activated CRAC Effectors through Designed Intramolecular Inhibition

**DOI:** 10.1021/acssynbio.2c00151

**Published:** 2022-07-08

**Authors:** Vid Jazbec, Roman Jerala, Mojca Benčina

**Affiliations:** † Department of Synthetic Biology and Immunology, National Institute of Chemistry, Hajdrihova 19, SI-1001 Ljubljana, Slovenia; ‡ EN-FIST Centre of Excellence, Trg Osvobodilne fronte 13, SI-1000 Ljubljana, Slovenia; § Interfaculty Doctoral Study of Biomedicine, University of Ljubljana, SI-1000 Ljubljana, Slovenia

**Keywords:** STIM1, Orai, TEV protease, PPV protease, calcium signaling, coiled-coil peptides

## Abstract

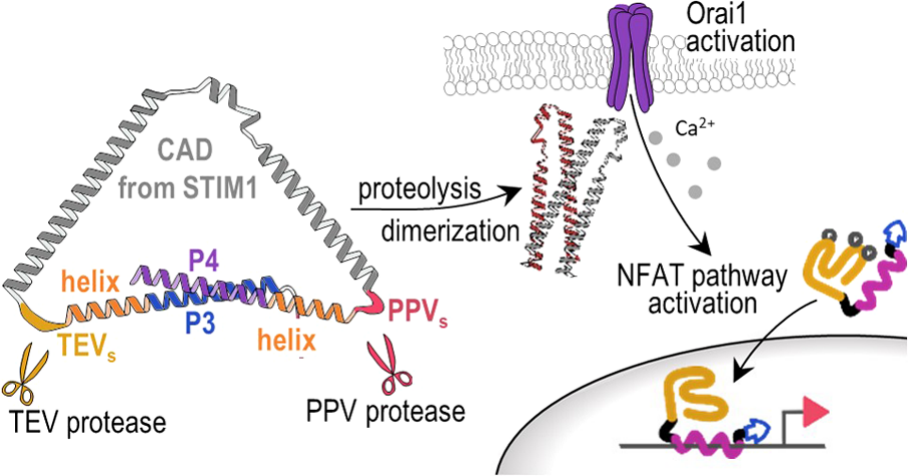

Highly regulated intracellular calcium entry affects numerous cellular physiological events. External regulation of intracellular calcium signaling presents a great opportunity for the artificial regulation of cellular activity. Calcium entry can be mediated by STIM proteins interacting with Orai calcium channels; therefore, the STIM1–Orai1 pair has become a tool for artificially modulating calcium entry. We report on an innovative genetically engineered protease-activated Orai activator called PACE. CAD self-dimerization and activation were inhibited with a coiled-coil forming peptide pair linked to CAD via a protease cleavage site. PACE generated sustained calcium entry after its activation with a reconstituted split protease. We also generated PACE, whose transcriptional activation of NFAT was triggered by PPV or TEV protease. Using PACE, we successfully activated the native NFAT signaling pathway and the production of cytokines in a T-cell line. PACE represents a useful tool for generating sustained calcium entry to initiate calcium-dependent protein translation. PACE provides a promising template for the construction of links between various protease activation pathways and calcium signaling.

## Introduction

Intracellular free calcium ions (Ca^2+^) serve as secondary messengers that are essential for the correct functioning of several cell processes, from short-term muscle contraction to long-term changes in gene expression;^1^ if their temporal concentration within cells is not carefully controlled, various severe cell dysfunctions can occur. Ca^2+^/calcineurin/nuclear factor of activated T-cells (NFAT) signaling modulates immune response with the transcriptional regulation of cytokines and chemokines in immune cells; NFAT activity is critical for Th cell differentiation.^2^ An assembly of plasma membrane- and endoplasmic reticulum (ER)-based ion channels and transporters orchestrates the concentration of intracellular Ca^2+^. The duration and amplitude of the Ca^2+^ signal produced by opening these channels govern a Ca^2+^-dependent signaling response.^3^

Store-operated Ca^2+^ entry is a Ca^2+^ current that responds to the depletion of Ca^2+^ in the ER and is facilitated by the ER membrane resident sensor molecule stromal interaction molecule 1 (STIM1) and the plasma membrane Ca^2+^ release-activated Ca^2+^ (CRAC) channel Orai1. Depletion of Ca^2+^ in the ER leads to conformational changes in the STIM1 ER domain, resulting in the exposure of the effector domain of the molecule, the CRAC activating domain (CAD, amino acid residues 342–448).^4,5^ This domain binds to the intercellular C-terminus of the Orai1 channel and renders the channel open.^6^ Studies have shown that CAD alone or even its truncated version STIM/Orai activating region (amino acid residues 344–442) is sufficient to fully activate the channel.^7^ The monomeric CAD forms a distinct letter R shape, which consists of four α-helical regions. Structural and mutagenic analysis of the domain has also shown that CAD dimerization is vital to Orai activation, and identified important residues enabling dimerization.^8^ In the same study, it has also been shown that the mutation of residues L347, W350, and L351 of α helix 1 (CC2) and W430, I433, and L436 of α helix 4 (CC3), which form interactions between the helices, hinders the ability to activate Orai1.

The designed mobilization of Ca^2+^ to activate transcriptional activity has already been used in the field of optogenetics, in which light-sensitive protein domains serve as switches to activate the effector domain from the ER calcium sensor protein STIM1^9−11^ or the CRAC channel Orai1.^12^ The role of Ca^2+^ as a transcriptional regulator has been explored in the combination with NFAT as a Ca^2+^-regulatory domain.^9,13,14^ The activation of the NFAT signaling pathway depends on long Ca^2+^ signals that keep the dephosphorylated NFAT in the cell nucleus.^15^ As CRAC current is used to maintain such signals,^16^ its components are promising tools for the activation of the artificial NFAT pathway. To date, the modulation of the STIM1 pathway has involved the use of intact STIM1 domain architecture. We seek to explore the possibility of regulating the intermolecular contacts of the CAD domain. Proteolytic activity has already been described as a fast and modular output of a synthetic circuit.^17,18^ Proteolytic cleavage is an irreversible process, and the proteolytically processed effectors could facilitate the sustained signals. Although light as in optogenetics provides good regulation of effectors’ activity, for sustained activity a prolonged exposure to light is required.^10^ Therefore, we reasoned that designing inactive variants of the CAD domain that could help regain its biological activity through proteolytic cleavage might be possible.

In this study, we developed the proteolytically activated CRAC effector (PACE), a genetically engineered mediator between proteolytic activity and calcium influx. The coiled-coil peptide pair P3:P4,^19−21^ genetically fused to a CAD activating domain, was introduced to inhibit CAD activity. A tobacco etch virus protease cleavage site (TEV_s_) or a plum pox virus protease cleavage site (PPV_s_) introduced between CAD and a coiled-coil segment enabled the activation of PACE by a defined protease that could be chemically regulated. The chemically reconstituted split protease activated PACE and triggered the long sustained Ca^2+^ entry and transcriptional activity of NFAT. Thus, PACE represents a useful tool for regulating Ca^2+^ entry and initiating physiologically relevant NFAT activation, which was demonstrated with the expression of cytokines in Jurkat T-cells.

## Results

### Engineered Orai Activators

**Figure 1:**
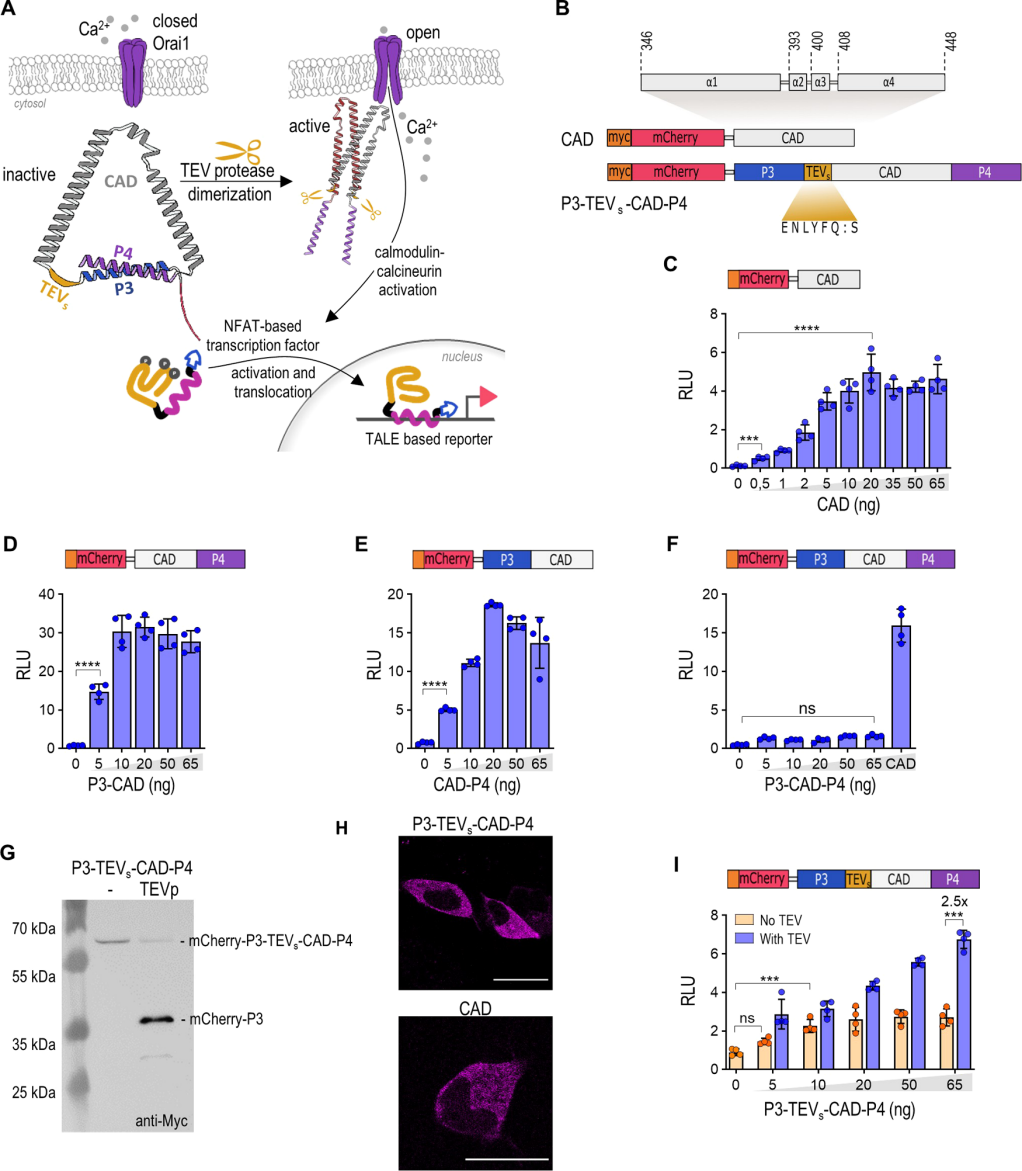
Coiled-coil P3:P4 peptide pair inhibits CAD. (A) Scheme of an engineered activator of Orai. The P3 and P4 peptides form a coiled-coil that prevents the self-activation of CAD. The protease cleaves the TEV_s_ between P3 and CAD and enables the dimerization of CAD into an active form. The protease-activated CAD activates Ca^2+^-dependent ion channel Orai. The Ca^2+^ influx activates the synthetic NFAT-based transcription factor, translocation in the nucleus, and expression of a gene of interest. (B) Engineered Orai activator. The structure of the CAD domain from STIM1 is highlighted above. Legend: red and orange, mCherry with Myc-tag; blue and violet, P3 and P4 coiled-coil peptides; gray, CAD; dark yellow, TEV_s_. (C–E) Constitutive activity of the engineered NFAT-based transcription factor in the presence of CAD (C), P3-CAD (D), or CAD-P4 (E) effector in HEK293 cells. (F) The inactive P3-CAD-P4 effector fails to activate the NFAT-based transcription factor in HEK293 cells. (G) Immunodetection of the uncleaved and cleaved P3-TEV_s_-CAD-P4 variant in HEK293T cells using anti-Myc antibodies. (H) Localization of mCherry-tagged P3-TEV_s_-CAD-P4 in HEK293 cells. The scale bar represents 20 μm. (I) The P3-TEV_s_-CAD-P4 effector exhibits the activation of an engineered NFAT-based transcription factor in the presence of TEV protease in HEK293T cells. One day after transfection, the HEK293T cells were lysed, and reporter activity was measured. The amounts of transfected plasmids are listed in Table S1. The bars represent the mean ± s.d.; *n* = 4 biologically independent cell cultures. Statistical significance and fold change are indicated above the bars. Statistical analyses and the corresponding *p*-values are listed in Table S5.

ER-transmembrane STIM proteins are important regulators of Orai channels. STIM1 is composed of an ER-Ca^2+^ sensing domain, transmembrane helix, cytosolic CAD domain inhibited by an inhibitory domain composed of three alpha-helices, and an unstructured lysine-rich C-terminal domain.^22^ A Ca^2+^-dependent structural rearrangement of the STIM1 dimer exposes the CAD domain that activates Orai. A CAD domain alone tends to bind and open Orai channels; therefore, we used it as a template to design an engineered Orai activator. The binding of alpha-helices α1 (CC2) and α4 (CC3) and the subsequent dimerization of the domain are recognized to be crucial for the ability of CAD to bind to the channel.^23^ To prevent spontaneous CAD dimerization and activation, we used intramolecular P3 and P4 coiled-coil forming peptides as steric hindrances to force CAD into an inactive conformation.^24^ P3 peptide was fused to the N-terminus of CC2, and P4 was added to the C-terminus of CC3 (Figure 1A). P3 and P4 peptides form a rigid parallel coiled-coil dimer that prevents spontaneous CAD dimerization. For the activation of CAD, the protease recognition site was introduced between the coiled-coil forming peptide and the CAD domain. The effect of the engineered Orai activators was tested using a reporter plasmid with 10 repeats of the transcription activator-like effector (TALE) binding site upstream of a minimal promoter (10TALE_Pmin-fLuc) expressing reporter firefly luciferase and a plasmid expressing an engineered NFAT-based transcription factor mNFAT:TALE:VP16:KRΦ^13^ (Figure 1A). The activity of transcription factor depends on the retention in the nucleus; therefore, constructs capable of generating sustained Ca^2+^ influx, which promote dephosphorylation of the transcription factor, are favored. The CAD domain was N terminally linked to mCherry;^25^ we added a Myc tag at the N terminal of mCherry to enable protein visualization (Figure 1B).

To adjust for the best response, we titrated the amount of CAD-coding plasmid (Figure 1C) and used 20 ng of CAD in subsequent experiments, as this concentration, compared with A21387 calcium ionophore, achieved full activation (Figure S1). The addition of a P3 peptide to the N terminus (P3-CAD) or of P4 to the C terminus of CAD (CAD-P4) did not prevent the spontaneous activation of Orai and the transcriptional activation of mNFAT:TALE:VP16:KRΦ (Figure 1D, 1E). However, when combined in the same polypeptide chain, P3 and P4 formed a stable intramolecular coiled-coil structure. This motif functions as a restraint that separates the ends of α helices 1 and 4 of the CAD domain. The P3-CAD-P4 construct, therefore, lost its ability to trigger the Ca^2+^-dependent transcriptional activation of a reporter (Figure 1F), thus confirming the feasibility of our concept. To introduce the controlled activation of the engineered Orai activator, we added seven amino acid-long TEV_s_ between helix P3 and the CAD domain (Figure 1B) and confirmed the cleavage of the engineered CAD with TEV protease (Figure 1G). The P3-TEV_s_-CAD-P4 construct was localized in the cytosol similar to wt CAD (Figure 1H). Cotransfection of a plasmid expressing TEV protease in cells expressing P3-TEV_s_-CAD-P4 and mNFAT:TALE:VP16:KRΦ transcription factor activated the expression of a reporter (Figure 1I). The transcriptional activity in cells with coexpressed TEV protease was 2.5 times higher than that in cells with no protease. However, the expression of the reporter was also detected in cells with no protease present, indicating the self-activation of the engineered Orai activator, which might be due to the insertion of TEV_s_ that added a flexible linker between P3 and CAD.

### Proteolytically Activated Activators of Orai

**Figure 2:**
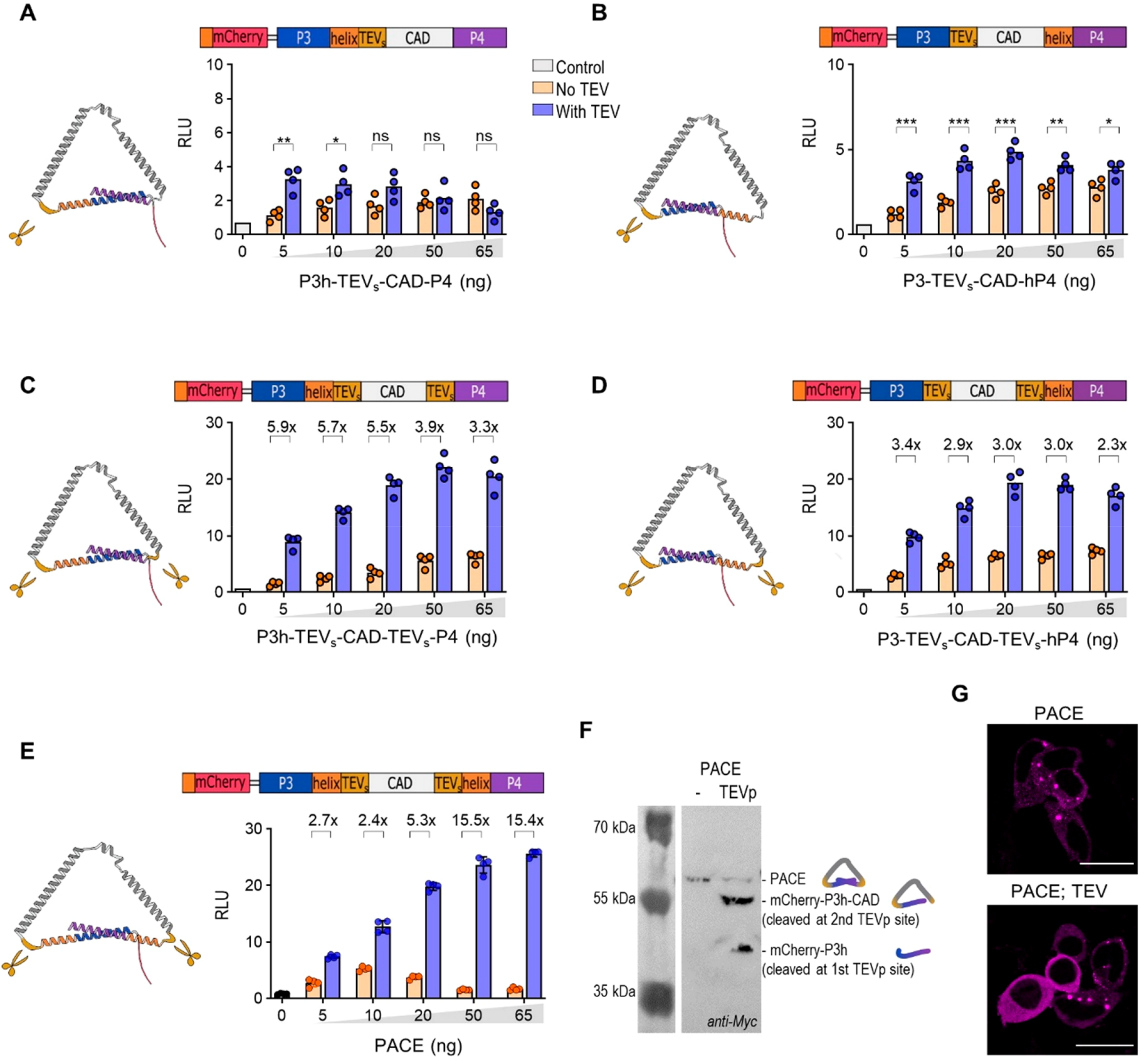
Proteolytically activated CRAC effector variants. (A–D) Transcriptional activity of the engineered mNFAT-based transcription factor in the presence of CRAC activators: (A) P3h-TEV_s_-CAD-P4, (B) P3-TEV_s_-CAD-hP4, (C) P3h-TEV_s_-CAD-TEV_s_-P4, and (D) P3-TEV_s_-CAD-TEV_s_-hP4 in HEK293T cells. (Left) Structural model of the effector. For clarity, mCherry was not depicted. Schemes depict proteolytically activated CRAC effector variants. The rigid helix was inserted between the coiled-coil peptides P3 and P4 and the TEV_s_ site. Legend: red, mCherry with Myc-tag; blue and violet, P3 and P4 coiled-coil peptides; gray, CAD; dark yellow, TEV_s_; orange, rigid helix. (E) Protease-induced transcriptional activity of the NFAT-based transcription factor in the presence of PACE in HEK293T cells. (F) Immunostaining of HEK239T cells expressing PACE variants in the absence or presence of TEV protease with anti-Myc antibodies. (G) Localization of PACE in HEK293 cells. The scale bar represents 20 μm. One day after transfection, the cells were lysed, and reporter activity was measured. The bars represent the mean ± s.d.; *n* = 4 biologically independent cell cultures. Statistical significance and fold change are indicated above the bars. Statistical analyses and the corresponding *p*-values are listed in Table S5. The amounts of transfected plasmids are listed in Table S1.

To extend the distance between α1 and α4 helices and thus prevent the spontaneous formation of active CAD, the peptide EEEEKKKKEEEEKK with a high helical propensity^26^ was added between the coiled-coil peptide and the CAD helix. The rigid helix-forming peptide was added C-terminally of P3 or N-terminally of P4, forming P3h-TEV_s_-CAD-P4 or P3-TEV_s_-CAD-hP4, respectively (Figure 2A, 2B). While the addition of a rigid helix in the construct P3h-TEV_s_-CAD-P4 reduced the background signal, it also attenuated activation with a TEV protease (Figure 2A; S2A). No improvement in activity was determined when a rigid helix was inserted between CAD and P4 peptide in P3-TEV_s_-CAD-hP4 (Figure 2B; S2B), although the TEV protease effectively cleaved the P3 peptide from CAD (Figure S3).

To preserve a low background signal and augment the activation with a protease, we added the second protease cleavage site between the CAD domain and the P4 coiled-coil forming peptide (Figure 2C, 2D). This indeed improved the fold activation between the proteolytically activated and inactive systems (Figure 2C, S2C; P3h-TEV_s_-CAD-TEV_s_-P4; Figure 2D, S2D; P3-TEV_s_-CAD-TEV_s_-hP4). However, it also increased the background signal. A rigid helix was then inserted at the N-terminal site of the P4 peptide, providing a P3h-TEV_s_-CAD-TEV_s_-hP4 construct, which we named PACE (Figure 2E, S2E). The TEV protease-cleaved PACE, as shown by Western blot, with two bands presenting the product, cleaved at the first or second TEV_s_ (Figure 2F).

PACE was expressed in the cytosol, forming bright spots (Figure 2G); unexpectedly, we also observed the colocalization of inactive PACE with overexpressed Orai1 (Figure S4A), as it is characteristic of CAD.^25,27^ The observed bright spots of PACE, which were probably the results of the intermolecular binding of coils P3 and P4, were also detected in the presence of TEV protease, although the removal of mCherry:P3 and P4 was expected after processing with protease (Figure 2H, S4B). On the basis of the optimization of constructs combining CC segments, a single α-helical segment, and a protease cleavage site, we managed to obtain a PACE construct characterized by a low background signal and as high as 15.5-fold activation in the presence of a TEV protease (Figure 2E). This construct was used in subsequent experiments.

### Switched Activation of PACE and Influx of Calcium

**Figure 3:**
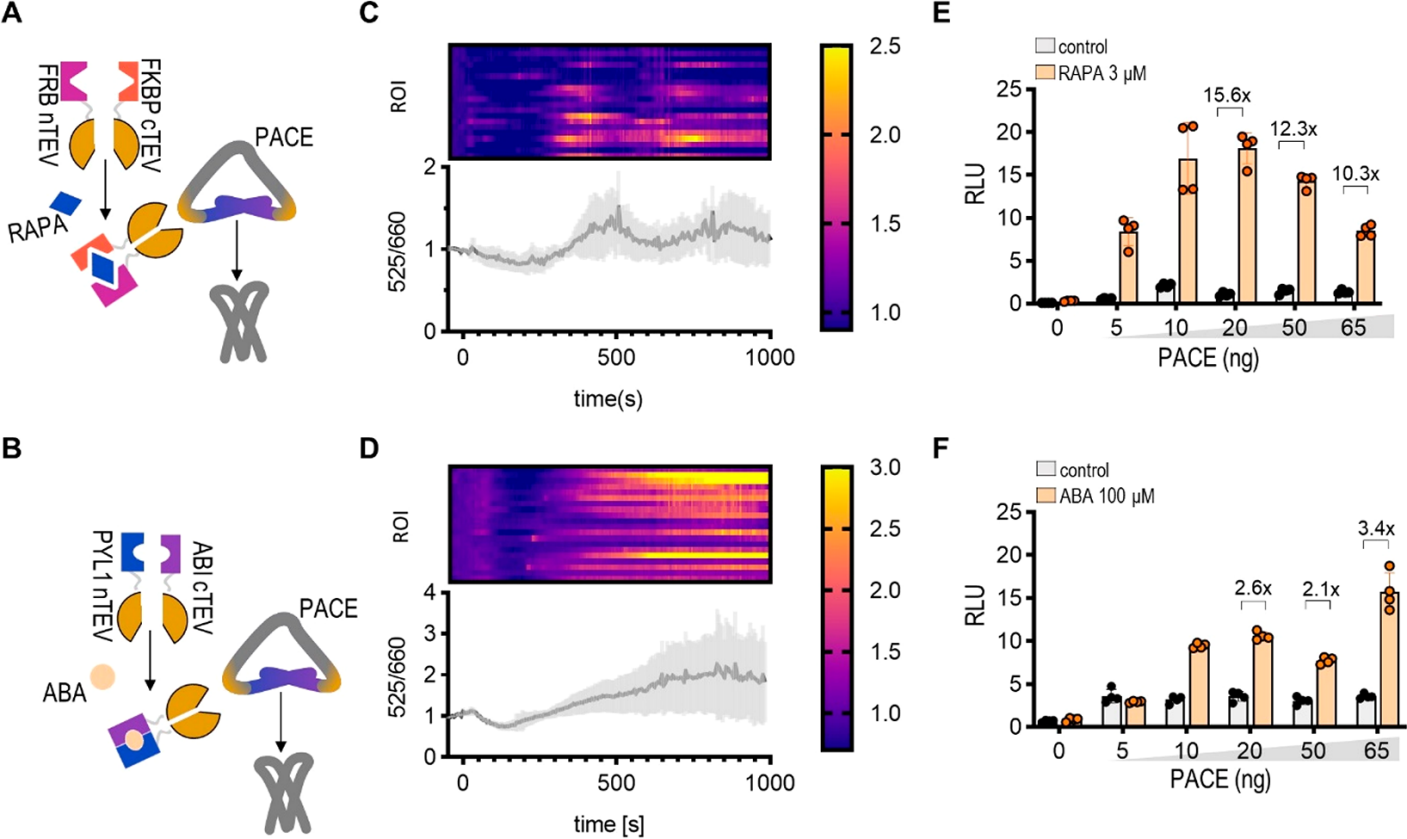
Kinetics of chemically regulated PACE activation through a split TEV protease. (A, B) Scheme of the design of PACE activation with RAPA (A) or ABA (B). The split TEV protease is activated with RAPA or ABA, and the active TEV protease cleaves off the intramolecular inhibitory P3:P4 coiled-coil. (C, D) The Ca^2+^ influx after the addition of RAPA (C) or ABA (D) was observed under a confocal microscope using CalRed dye. Twenty regions of interest with one to five cells in which we could identify mCherry fluorescence were selected, and the ratio of their fluorescence at 525 and 660 nm is shown as a time-dependent graph and heat map. A time-lapse video of a region for each experiment is available in Supplementary Videos S1 and S2. The amounts of transfected plasmids for all microscopy experiments are listed in Table S3. (E, F) HEK293T cells expressing PACE and the split TEV protease were induced by 3 μM RAPA (E) or 100 μM ABA (F). Six hours later, firefly luciferase reporter activity was measured. The amounts of transfected plasmids for all luciferase experiments are listed in Table S1. One day after transfection, the cells were lysed, and reporter activity was measured. The bars represent the mean ± s.d.; *n* = 4 biologically independent cell cultures. Fold change is indicated above the bars. Statistical analyses and the corresponding *p*-values are listed in Table S5.

In our effort to produce a switchable modulator of CRAC, we used a split TEV protease, which consists of N- and C-terminal fragments of the TEV protease fused to either FKBP and FRB (the heterodimerization of which is inducible with rapamycin [RAPA]) or to ABI and PYL1 (the heterodimerization of which is inducible with abscisic acid [ABA])^17^ (Figure 3A, 3B). Efficient chemical-regulated activation mediated by the PACE effector was achieved by stimulation with RAPA or ABA, which spontaneously passes the cell membrane and enables the split TEV protease to reconstitute and cleave recognition sites on PACE. Active PACE then triggered an influx of Ca^2+^ and Ca^2+^-dependent transcription.

One of the important advantages of chemically regulated Ca^2+^ influx regulators is their potential to trigger fast responses. The kinetics of Ca^2+^ influx after PACE activation were monitored in vivo in HEK293 cells using the calcium indicator CalRed. While ionophore produced an immediate and lasting influx of calcium into the cytosol compared with untreated cells (Figure S5A, S5B), RAPA or ABA induced a smaller but significant Ca^2+^ influx 300 s after the addition of a chemical inductor (Figure 3C, 3D). No cytotoxicity of ABA or RAPA in combination with PACE was observed on HEK293T cells (Figure S6A, S6B).

Next, we examined whether ABA or RAPA could be used to induce the activation of PACE and the transcriptional activation of the synthetic Ca^2+^-responsive NFAT-based transcription factor. The induction of reporter expression was detected only in the presence of PACE and RAPA or ABA (Figure 3E, 3F; Figure S6C, S6D, comparison with CAD), with as high as a 15-fold increase with the addition of 3 μM RAPA and a 3-fold increase with the addition of ABA. The addition of gadolinium chloride blocked transcriptional activation (Figure S6E, S6F), demonstrating that the PACE effector activated calcium-dependent ion channels, most likely through Orai.

### Construction of a Logic OR Gate-PACE Effector with Dual Regulation

**Figure 4:**
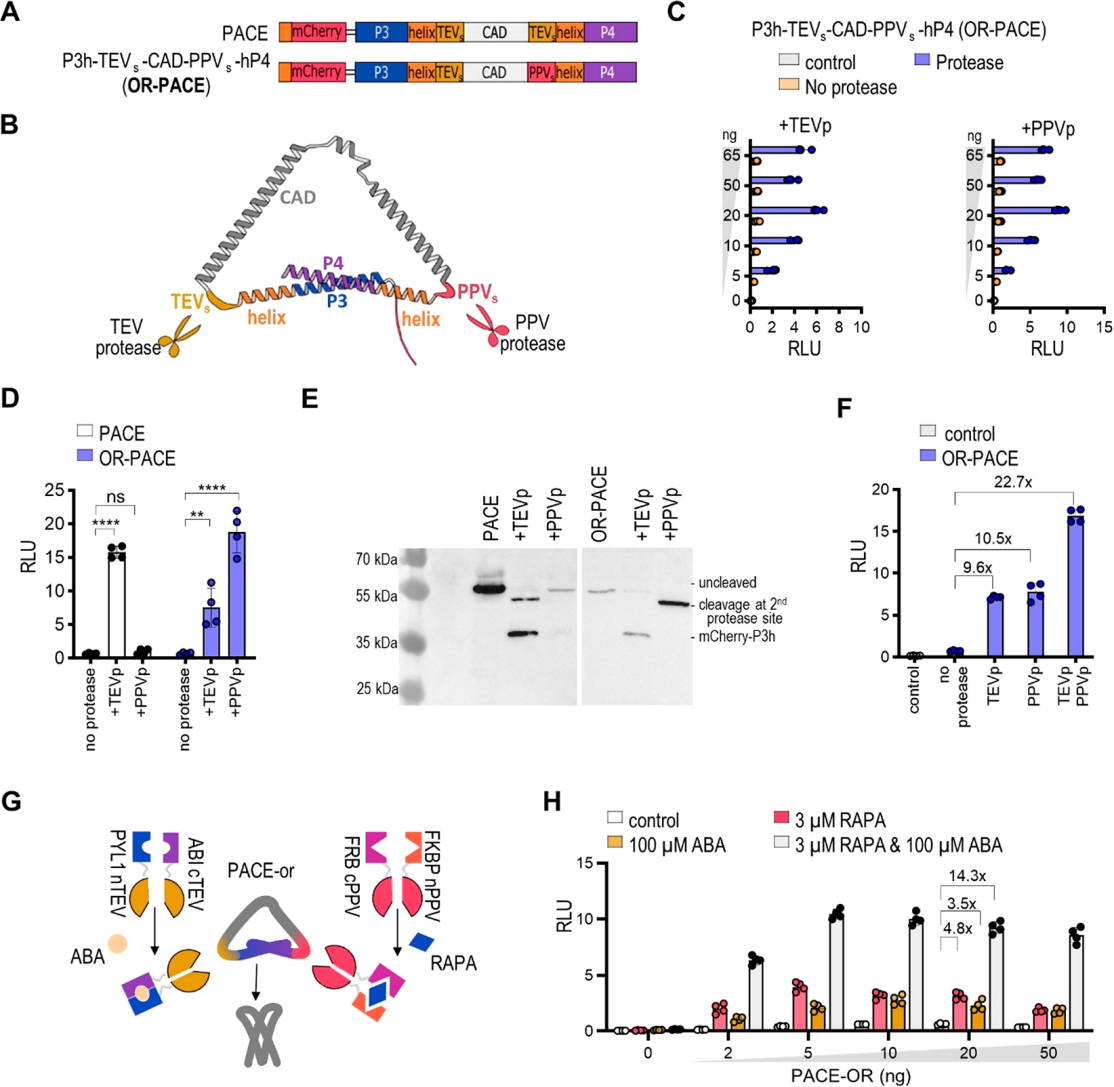
PACE effector with dual regulation. (A) PACE variants with various combinations of TEV_s_ and PPV_s_ protease recognition sites. (B) The scheme depicts proteolytically activated CRAC effector variant P3h-TEV_s_-CAD-PPV-hP4, with TEV_s_ and PPV_s_ depicted in yellow and red, respectively. (C, D, F) Dual-luciferase assay of PACE variants in the presence of TEV_s_ and/or PPV_s_. (E) Immunodetection of PACE without and in the presence of coexpressed TEV or PPV protease stained with anti-Myc antibodies. (G) Scheme of the combination of two chemically induced split proteases and PACE-OR. (H) Dual-luciferase assay of PACE-OR in the presence of split TEV_p_ and split PPV_p_. Folds are presented for the same concentration of plasmid as in (F). The amounts of transfected plasmids for all luciferase experiments are listed in Table S1. One day after transfection, the cells were lysed, and reporter activity was measured. The bars represent the mean ± s.d.; *n* = 4 biologically independent cell cultures. Statistical significance and fold change are indicated above the bars. Statistical analyses and the corresponding *p*-values are listed in Table S5.

TEV protease is commonly used in synthetic biology; it does not interfere with cellular processes in human or bacterial cells because of its high specificity for the cleavage site.^28^ To expand the toolbox of PACE effectors, the TEV_s_ recognition site was changed to a PPV_s_ cleavage site (NVVVHQA). The lengths of TEV_s_ and PPV_s_ are the same, but the proteases are orthogonal.^29^ Three additional PACE variants were prepared, and they contained PPV_s_ at the first, second, or both cleavage sites (Figure 4A, 4B, S7A). Surprisingly, we observed significant differences in the background activation of the PACE variants. No inhibition of CAD activity was observed for unactivated PACE variants with the PPV_s_ positioned between P3 and CAD (Figure S7B, S7C). On the other hand, a type of protease cleavage site between CAD and P4 peptide had no impact on background activity (Figure 4C, 4D). While the second site enabled the activation of PACE with either protease, the substitution of the first site for the PPV_s_ rendered PACE constitutively active. The PPV and TEV proteases cleaved the PACE variants, generating predicted fragments (Figure 4E, S7D). We also assessed the activity of the PACE with two different sites in combination with one or both proteases present. Although a single cleavage was sufficient to promote Ca^2+^ signaling (10-fold for either TEV or PPV protease), the removal of both inhibitory coiled-coil peptides from CAD renders additional activation to CAD about 23-fold (Figure 4F, S7E).

Next, the P3h-TEV_s_-CAD-PPV-hP4 (PACE-OR) was combined with the ABA-inducible split TEV_p_ and RAPA-inducible split PPV_p_ proteases (Figure 4G, 4H). The addition of ABA or RAPA alone or in combination induced the corresponding cleavage and activation of PACE-OR. However, the 14-fold increase in reporter expression was detected with the addition of both inducers together, compared to a 4.8- and 3.5-fold increase with the addition of only ABA or RAPA, respectively.

### PACE-Regulated IL2 and TNFα Production in Jurkat T-Cells

**Figure 5:**
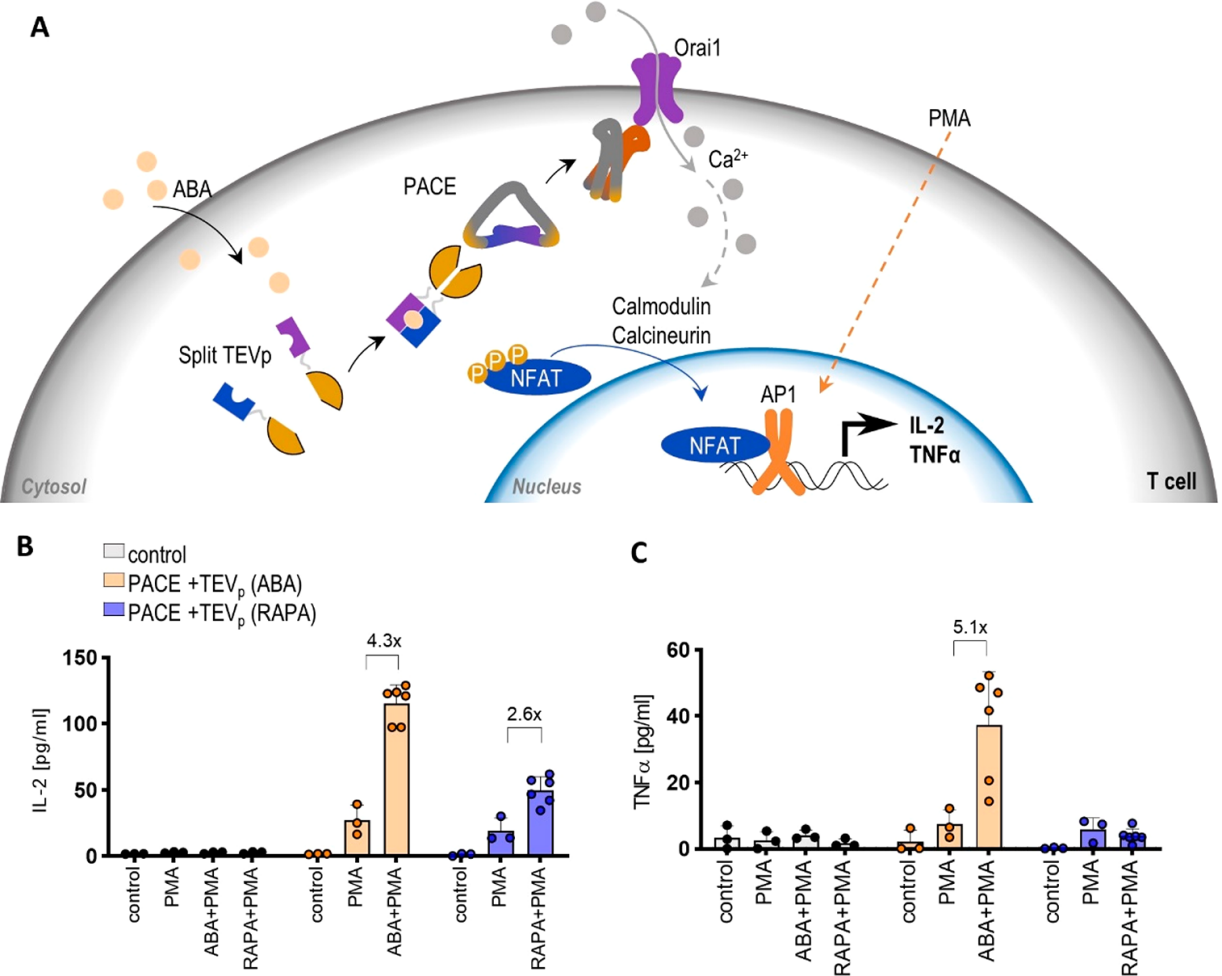
Induction of T-cell activation by a chemical signal using PACE-mediated Ca signaling. (A) Entry of ABA into cytosol enables the reconstitution of functional TEV protease, which cleaves PACE at its recognition sites. The cleaved PACE dimerizes and opens the Orai1 Ca^2+^ channel. An increase in cytosolic Ca^2+^ activates calmodulin and thus calcineurin, which dephosphorylates NFAT, enabling it to enter the nucleus. Interleukin 2 promoter requires an additional transcription factor for NFAT, AP-1,^33^ which is achieved by the downstream effect of the addition of PMA. (B, C) ELISA assay for IL-2 (B) and TNFα (C) production in Jurkat T-cells expressing PACE and the split TEV protease. Production was induced with PMA and 3 μM RAPA, PMA, or 100 μM ABA. The amounts of transfected plasmids for all ELISA experiments are listed in Table S4. One day after transfection, the cells were centrifuged, and the supernatant was taken for ELISA. The bars represent the mean ± s.d.; *n* = 3 or 6 biologically independent cell cultures. Fold changes are indicated above the bars. Statistical analyses and the corresponding *p*-values are listed in Table S5.

To confirm Ca^2+^ influx with PACE for the regulation of a physiologically relevant function, we aimed to design a system that triggers the activation of T-cells mediated via Ca^2+^ and regulated by chemical signals. For this purpose, we introduced PACE and chemically regulated protease into T-cells, in which the activation of an NFAT signaling pathway should be triggered via Ca^2+^ influx. We electroporated plasmids expressing PACE and the split TEV protease into the T-cell line. Jurkat T-cells are a well-established cell line,^30^ a model for T-cell activation, which enabled us to test the production of interleukin 2 (IL-2), a versatile cytokine responsible for T-cell growth, the differentiation of regulatory T-cells, and the mediation of activation-induced cell death.^31^ Transcription of IL-2 is NFAT and AP-1 dependent; therefore, an additional coactivator, phorbol 12-myristate 13-acetate (PMA), is needed to induce upregulation of IL-2 transcription.^32,33^ The electroporated Jurkat T-cells were subjected to PMA and ABA or RAPA stimulation (Figure 5A). ABA and RAPA, in combination with PMA, induced the synthesis of IL-2 (Figure 5B); however, ABA-induced IL-2 production was higher than that induced by RAPA. We also observed the production of tumor necrosis factor-alpha (TNFα), which is regulated by NFAT as the key transcription factor (Figure 5C); TNFα is an important cytokine involved in immune response.^34^ Taken together, PACE represents a tool for generating sustained Ca^2+^ entry to initiate NFAT dependent protein translation.

## Discussion

We have designed an engineered proteolysis-based Orai activator named PACE. The PACE activator domain CAD originates from STIM1 and is composed of CC2 and CC3 helices that spontaneously associate and dimerize.^8^ To prevent spontaneous CAD activation, coiled-coil forming peptides P3 and P4 were introduced. P3 and P4 form a stable, rigid coiled-coil dimer^20^ that restrains the formation of an active CAD by forming an intramolecular dimer that separates the N- from the C-terminus of CAD. Insertion of a protease cleavage site between CAD and an inhibiting coiled-coil enabled the controlled activation of PACE. Because of the nature of the proteolytic activity, which irreversibly activates the protein, PACE can induce long, sustained Ca^2+^ currents that activate the Ca^2+^/calcineurin/NFAT signaling pathway either for engineered NFAT-based transcription factors or native NFAT. To increase the signal-to-background ratio, coiled-coil forming peptides were extended with rigid helix forming peptides.

The inactive PACE is mainly a cytosol-based protein with minor interactions with membrane-based Orai channels. While designed for intramolecular interactions, P3 and P4 coiled-coil forming peptides could also make intermolecular connections, which can likely explain the higher-order structures of PACE, detected as bright spots. Despite the formation of higher-ordered structures, the desired regulation was nevertheless achieved, and the activity of PACE was not significantly reduced, nor did such interactions increase unwanted Orai activation, which means that such assemblies still inhibit CAD activity. The use of coiled-coil peptides with *K*_d_ in the nanomolar range (e.g., N5:N6, *T*_m_ = 76 °C)^19^ did not improve the fold of activation (data not shown), which likely suggests multiple weak interhelical interactions.

PACE also provided a template for the variation of protease recognition sites. In addition to the TEV protease cleavage site, we tested the activation of PACE with an orthogonal PPV protease.^29^ Interestingly, the replacement of TEV_s_ at the first site between P3 and CAD with PPV_s_ reduced the signal-to-background ratio. On the other hand, the substitution of the second TEV_s_ site between CAD and P4 with PPV_s_ maintained a high signal-to-background ratio. The difference between the first and second cleavage sites probably occurred because the second cleavage site is flanked by a Gly-Pro-Gly and Gly-Ser-Gly peptide sequence, which enables substantial flexibility. The first cleavage site was retained without flanking peptides, as the TEV_s_ cleavage site sequence proved to be sufficient. By combining TEV_s_ at the first protease cleavage site and PPV_s_ at the second one, an effective “OR” logic gate was created with an added feature of a 2-fold higher signal when cleaved at both sites. This can be explained by the fact that for full activation of PACE, a cleavage at both sites, between P3 and CAD, and between CAD and P4, is necessary. During designing PACE, it became evident that activated PACE with two protease cleavage sites generates more efficient transcriptional activation probably due to more effective activation of Orai channels. Activation by two possible inputs enables the design of more complex synthetic biological circuits. This was exploited with the inducible activation of PACE-OR in combination with ABA or RAPA-induced TEV_p_ or PPV_p_ protease. As proteases can be activated in a variety of ways, PACE enables the existing protease-based circuits^17,18,35,36^ to be coupled with Ca^2+^ signaling networks of the cell.

With externally activated split proteases, we have determined the kinetics of PACE activation. Compared with the rapid Ca^2+^ influx triggered by ionophore, PACE activation is slower (8–16 min). The delay in Ca^2+^ influx can be attributed to the time required for RAPA or ABA entry and to the reconstitution of split protease reconstitution. The PACE activation time is comparable to the activation time of cyclic luciferase reporter using ABA or RAPA-inducible split TEV proteases.^17^ Compared to activation of optogenetic constructs by light, PACE response to activation is delayed also due to the lack of the polybasic region, which is present at the C-terminus of native STIM and anchor active CAD in the proximity of Orai channel.^11^ A lower amplitude of Ca^2+^ was observed for PACE compared with ionophore; however, this could be beneficial, as it causes less damage to cells and activates different cell responses.^37^ The selection and evaluation of PACE constructs were based on their ability to activate NFAT based signaling; therefore, the intracellular Ca^2+^ amplitude and the rate of Orai activation were not considered in the selection process as they were secondary to the sustainability of Ca^2+^ signals.

As the construction of PACE was based on its ability to activate NFAT-based transcription factors, we tested its ability to activate native NFAT pathways in T-cells. PACE activated NFAT in a T-cell line, as detected with the secretion of IL-2 and TNFα. The expression of IL-2 requires the presence of PMA-activating AP1 in addition to NFAT.^33^ TNFα expression, in which NFAT1 transcription is sufficient, was also enhanced with PMA.^38^ However, protein expression using PACE was comparable to the existing optogenetic CRAC activators in both luciferase assay^10^ and IL-2.^39^ In T-cells, the transcriptional activation of NFAT with RAPA was less effective than that with ABA because of the immunosuppressing abilities of the RAPA upstream of the AP-1 signaling pathway.^40^

Synthetic biology research, especially optogenetics, has developed a varied toolbox of calcium current effectors.^41^ Each of them, however, is a standalone element and does not allow integration into larger and more elaborate synthetic networks. The PACE constructs presented in this study and dual protease site variant PACE-OR bridge this gap between existing synthetic circuits that regulate protease activity and the calcium current, and they enable the construction of larger and more elaborate biological circuits. With the possible switching of protease cleavage sites, PACE provides a robust scaffold for coupling not only synthetic circuits but also any existing well-defined protease activity, such as caspases or cathepsins, to calcium influx.

## Materials and Methods

### Cloning and Plasmid Construction

Orai1-GCaMP6f and mCherry-CAD were gifts from Michael Cahalan (Addgene plasmid # 73564; http://n2t.net/addgene:73564; RRID: Addgene_73564; Addgene plasmid # 73566; http://n2t.net/addgene:73566; RRID: Addgene_73566, respectively). All plasmids were constructed using the Gibson assembly method, and their sequences are listed in Table S6.

### Cell Culture

The embryonic kidney HEK293T and HEK293 cell lines (ATCC) were cultured in Dulbecco’s modified Eagle’s medium (DMEM; Invitrogen) supplemented with 10% fetal bovine serum (Gibco) at 37 °C in a 5% CO_2_ environment. In experiments with GdCl_3_, Dulbecco’s modified Eagle’s medium without phosphate (DMEM; Invitrogen) supplemented with 10% fetal bovine serum (Gibco) was used. Jurkat cells were cultured in Roswell Park Memorial Institute 1640 Medium (RPMI; Invitrogen) supplemented with 10% fetal bovine serum.

### Transfection, Electroporation, and Stimulation

For dual luciferase assays, 2 × 10^4^ HEK293T cells were seeded per well in 96-well plates (Corning). For confocal microscopy experiments, 5 × 10^4^ HEK293T cells were seeded per well in an eight-well chamber slide (Ibidi). For Western blot experiments, 5 × 10^5^ HEK293T cells were seeded per well in six-well plates (Corning). At 50–70% confluence, HEK293T cells were transfected with a mixture of DNA and polyethylenimine (PEI, linear, Mw 25000; Polysciences, catalog no. 23966). Per 500 ng DNA, 6 μL of PEI stock solution (0.324 mg/mL, pH 7.5) was used. Twenty-four hours after transfection, the culture medium was replaced with a fresh medium, and the cells were stimulated with the indicated concentrations of ionophore (calcium ionophore A23187, Sigma-Aldrich), RAPA (Sigma-Aldrich), or ABA (Goldbio) for 6 h. Calcium ionophore A23187 was prepared as a 10 mM stock solution in DMSO, RAPA as 1 mM stock, and ABA as 50 mM stock. The phRL-TK plasmid (Promega), encoding the Renilla luciferase, was used as a transfection efficiency control in the luciferase experiments. An empty pcDNA3 plasmid (Invitrogen) was used to equalize the total DNA amounts under different experimental conditions.

Jurkat cells were electroporated using the Neon Transfection System at a concentration of 2 × 10^7^ cells/mL with three pulses of 1600 V using 100 μL tips.

### Dual Luciferase Assays

Cells were lysed 24 h after transfection or 6 h after induction of RAPA, ABA, or ionophore using 25 μL of 1 × Passive lysis buffer (Promega) per well. Firefly luciferase (fLuc) and Renilla luciferase (rLuc) activities were measured using the dual luciferase assay (Promega) on an Orion II microplate reader (Berthold Technologies). Relative luciferase units were calculated by normalizing fLuc to the constitutive rLuc in each sample.

### Immunoblotting

HEK293T cells (2 × 10^5^) were seeded in 12-well plates (Techno Plastic Products). The next day, at a confluence of 50–70%, the cells were transiently transfected with a mixture of DNA and PEI (8 μL PEI/1000 ng DNA). Forty-eight hours post-transfection, the cells were washed with 1 mL PBS and lysed in 100 μL 1 × Passive lysis buffer (Promega). The cells were lysed for 20 min on ice and centrifuged for 15 min at 14 000 rpm to remove cell debris. The total protein concentration in the supernatant was determined using the BCA assay.

Proteins from the supernatant were separated on 12% SDS-PAGE gels (200 V, 45 min) and transferred to a nitrocellulose membrane (350 mA, 60 min). Membrane blocking, antibody binding, and membrane washing were performed using an iBind Flex Western device (ThermoFisher) according to the manufacturer’s protocol. The primary antibodies were mouse-anti Myc (Cell Signaling Technology 2276; diluted 1:2000). The secondary antibodies were HRP-conjugated goat antimouse IgG, diluted 1:3000 (Jackson ImmunoResearch 115-035-003). The secondary antibodies were detected with an ECL Western blotting detection reagent (Super Signal West Femto; ThermoFisher) according to the manufacturer’s protocol.

### Confocal Microscopy

For the analyses of fluorescent protein-expressing cells, live cells were imaged 1 day after transfection. During microscopy, the cells were kept in a chamber at 37 °C. To maintain the physiological pH, 10 mM 4-(2-hydroxyethyl)-1-piperazineethanesulfonic acid (HEPES) pH 7.4 (from 1 M stock solution) was added to the media. Microscopic images were obtained using a Leica TCS SP5 inverted laser scanning microscope on a Leica DMI 6000 CS module equipped with an HCX Plane-Apochromat lambda blue 63× objective and a numerical aperture of 1.4 (Leica Microsystems). A 50 mW 405 nm diode laser was used for GCaMP6f and excitation (emission between 420 and 460 nm), and a 10 mW 543 nm laser was used for mCherry excitation (emission between 550 and 650 nm).

For calcium imaging, we used CalRed ratiometric dye (ATT Bioquest 20591), which was added to the cells for 1 h at a concentration of 10 g/mL before exchanging it for the usual medium with an addition of 10 mM HEPES. Microscopic timelapse images were obtained using a Leica TCS SP5 inverted laser scanning microscope on a Leica DMI 6000 CS module equipped with an HCX Plane-Apochromat CS 40× objective and a numerical aperture of 1.25 (Leica Microsystems). A 10 mW 488 nm laser was used for CalRed excitation (emission in the Ca^2+^ unbound state between 497 and 545 nm, emission in the Ca^2+^ bound state between 617 and 696 nm). Leica LAS AF software was used for acquisition, and ImageJ software (National Institute of Mental Health, Bethesda, USA) was used for image processing.

### ELISA

Jurkat cells were seeded into a 96-well plate 24 h after electroporation at a concentration of 1.5 × 10^6^ cell/mL with the addition of 50 ng/mL PMA (Sigma-Aldrich P1585), RAPA, ABA, or ionophore. After 24 h, the cells were centrifuged, and the supernatant was taken and used for ELISA for Il2 (IL-2 Human Uncoated ELISA Kit 50-246-332, Thermo Fisher Scientific) and TNFα (TNF alpha Human Uncoated ELISA Kit with Plates 88-7346-22, Thermo Fisher Scientific), which were conducted following the manufacturer’s instructions. Absorbance was measured with a Synergy Mx automated microplate reader (BioTek) at 450 and 620 nm using Gen5 software. Absorbance at 620 nm was used for background correction and was subtracted from the absorbance at 450 nm.

### Statistical Analysis

The data are presented as mean values ± s.d. of four independent biological repeats within the same experiment. Graphs and statistical analyses were prepared in GraphPad Prism 8. For the analysis of the activity of variants with different protease recognition sites, unpaired Student *t* test was used, with 0 ng of constructs serving as the control for comparison. For the analysis of protease-based constructs without active protease, one-way ANOVA was used with Dunnett’s multiple comparisons test as a post hoc analysis. The mean of each condition was compared with the mean of the control (0 ng of the construct added). For the analysis of the activity of the activated constructs, an unpaired Student *t* test was used between the same concentration of constructs in the presence and absence of active protease. Statistical analysis is depicted in Table S5.

## Supporting Information Available

Figure S1: Titration of CAD in HEK293T cells in the presence or absence of calcium ionophore A23187; Figure S2: Dual-luciferase assay of Orai activators compared with CAD; Figure S3: Immunostaining of CAD variants expressed in HEK293 cells in the absence or presence of TEV protease; Figure S4: Microscopic image of HEK293 cells expressing PACE and Orai1-GCaMP6f without and with TEV protease; Figure S5: Calcium influx after the addition of ionophore and the control; Figure S6: Viability of HEK293T cells transfected with plasmids expressing PACE without or with the split TEV protease treated with RAPA or ABA, and inhibition of Ca^2+^-dependent transcriptional activation with gadolinium chloride; Figure S7: PACE effector with dual regulation; Figure S8: ELISA assay for IL-2 and TNFα production in Jurkat T-cells expressing PACE and a split TEV protease; Table S1: Amounts of transfected plasmids for HEK293T cells for each well in 96-well plates; Table S2: Amounts of transfected plasmids for HEK293T cells for each well in 12-well plates used for Western blot analyses; Table S3: Amounts of transfected plasmids for HEK293 cells for each well in eight-well plates used for microscopy; Table S4: Amounts of electroporated plasmids for Jurkat cells for each 100 μL electroporation; Table S5: Statistical analysis; Table S6: List of constructs with the amino acid sequences used in this study (PDF)
Supplementary Video S1 (MP4)
Supplementary Video S2 (MP4)

## Abbreviations

ABA: abscisic acid
CC: coiled coil
CAD: CRAC activation domain
CRAC: Ca^2+^ release uptake current
ER: endoplasmic reticulum
fLuc: firefly luciferase
HEK293: human embryonic kidney
IL-2: interleukin 2
NFAT: nuclear factor of activated T-cells
PMA: phorbol 12-myristate 13-acetate
PPV: plum pox virus
RAPA: rapamycin
RLU: relative light units
rLuc: Renilla luciferase
STIM: stromal interaction molecule
TALE: transcription activator-like effector
TEV: tobacco etch virus
TNFα: tumor necrosis factor alpha.
