# Supplementary material for: Proteolytically Activated CRAC Effectors through Designed Intramolecular Inhibition
Source: ACS Synth Biol. 2022 Jul 8;11(8):2756–65. doi: 10.1021/acssynbio.2c00151 (PMC9396659; doi:10.1021/acssynbio.2c00151)
Supplement: Supplementary file 1 — sb2c00151_si_001.pdf [file sb2c00151_si_001.pdf]

Proteolytically activated CRAC effectors through designed intramolecular inhibition

Vid Jazbec[#,1,3], Roman Jerala[1,2,*], Mojca Benčina[1,2,*]

[1] Department of Synthetic Biology and Immunology, National Institute of Chemistry, Hajdrihova 19, SI-1001 Ljubljana, Slovenia
[2] EN-FIST Centre of Excellence, Trg Osvobodilne fronte 13, SI-1000 Ljubljana, Slovenia
[3] Interfaculty Doctoral Study of Biomedicine, University of Ljubljana

[#] First author
[*] Correspondence: roman.jerala@ki.si (RJ); mojca.bencina@ki.si (MB)

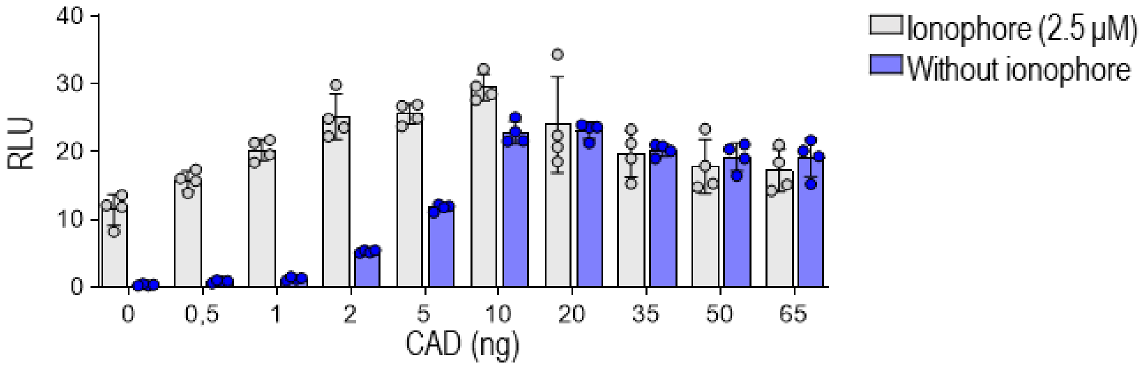

Fig. S1: Titration of CAD in HEK293T cells in the presence or absence of calcium ionophore A23187. HEK293T cells expressing CAD (0–65 ng), mNFAT:TALE:VP16:KRΦ, and the reporter were treated with or without ionophore (2.5 µM), and reporter expression was analyzed 6 h later. The amounts of transfected plasmids are listed in **Table S1**.

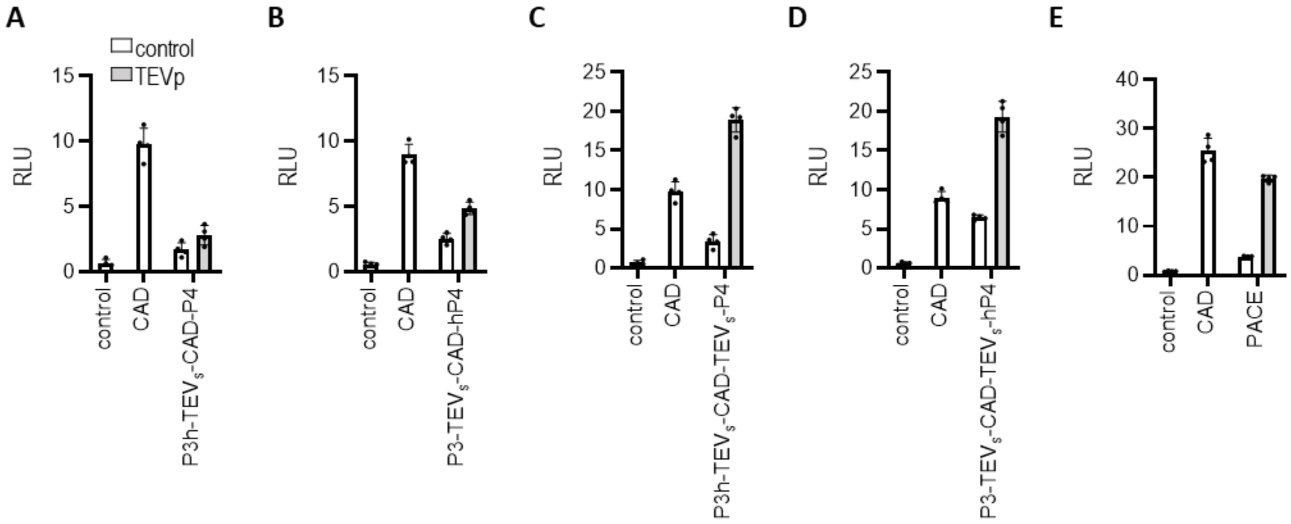

Fig. S2: Dual-luciferase assay at a concentration of 20 ng of Orai activators compared with CAD (both 20 ng). (**A–E**) correspond to the variants used in **Fig. 2**.

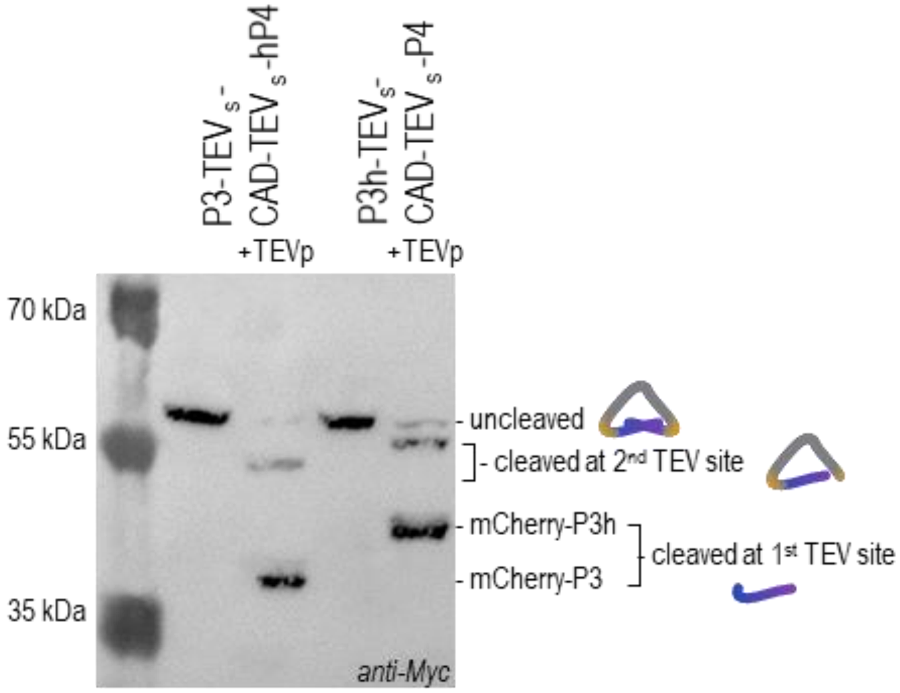

Fig. S3: Immunostaining of HEK239T cells expressing inhibited CAD variants in the absence or presence of TEV protease stained with anti-Myc antibodies.

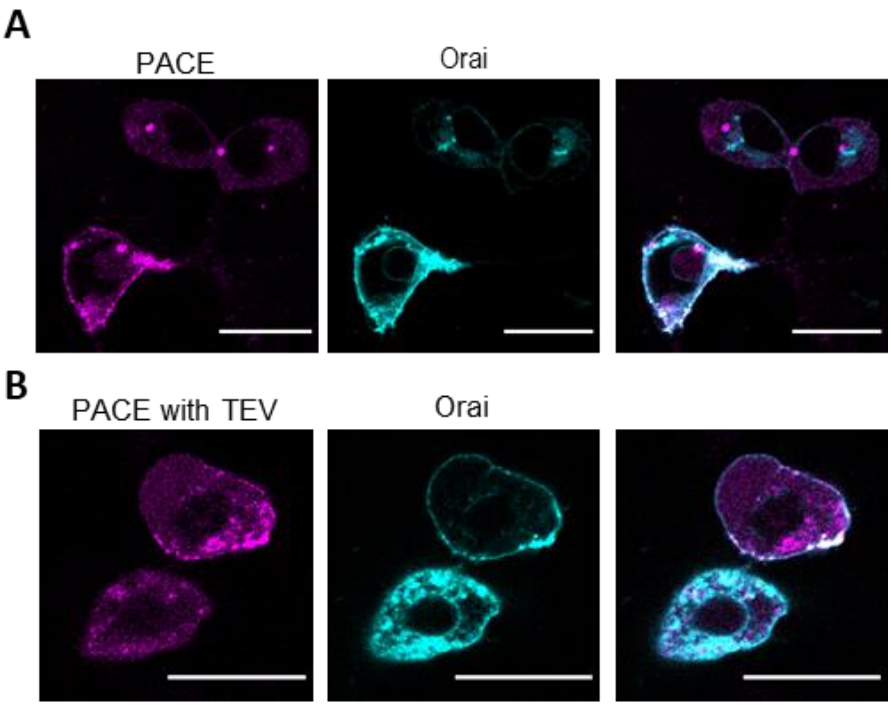

Fig. S4: Microscopic image of HEK293 cells transfected with plasmids expressing PACE and Orai1-GCaMP6f without (**A**) and with TEV protease (**B**). The scale bar represents 20 µm. *Note: No difference in localization was observed.*

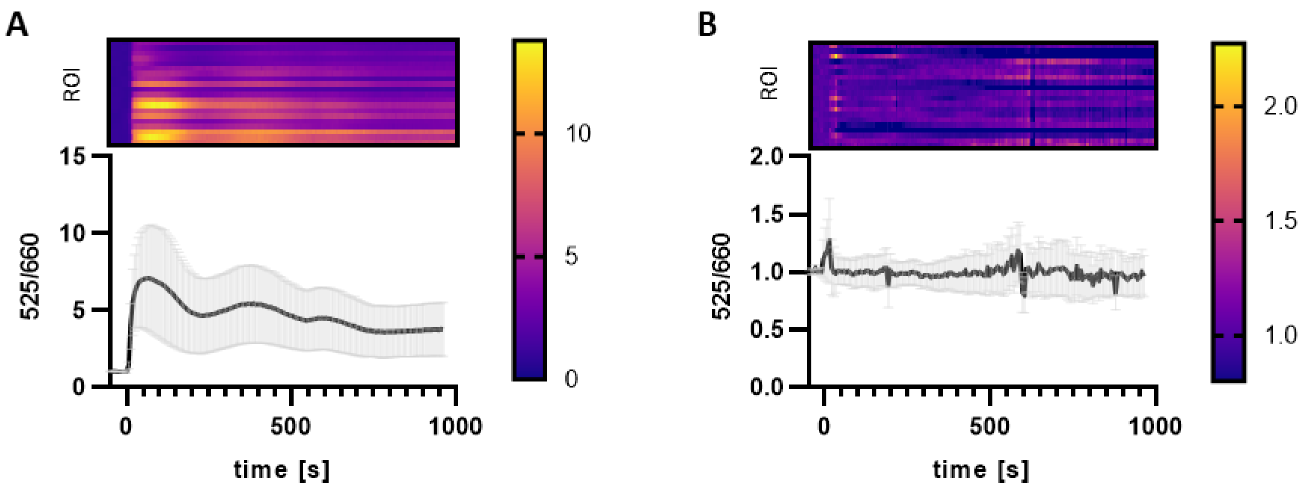

Fig. S5: Calcium influx after the addition of 2.5 µM ionophore (**A**) and the control (**B**). The change in calcium concentration was observed under a confocal microscope using CalRed.

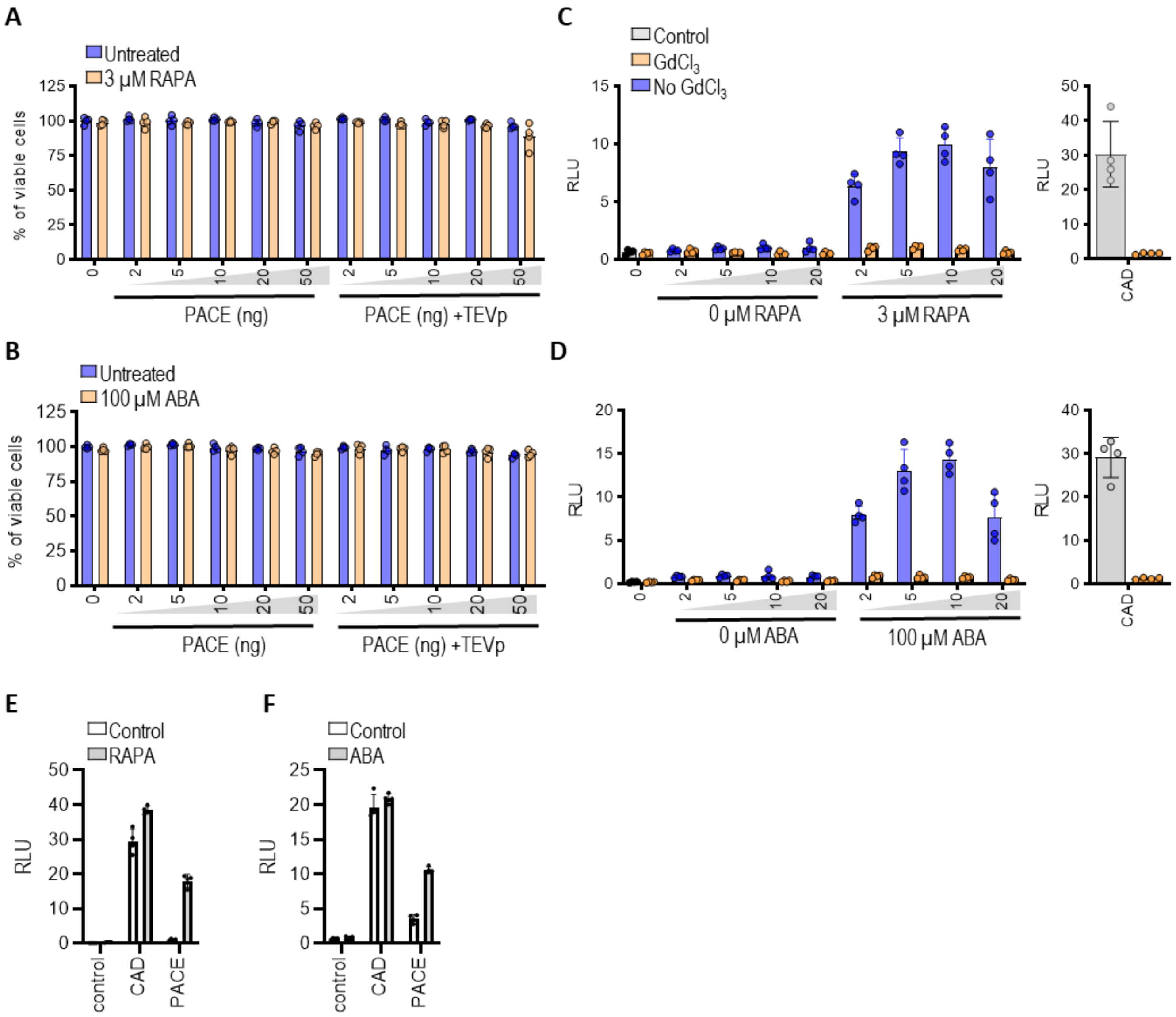

Fig. S6:
(**A, B**) Viability of HEK293T cells transfected with plasmids expressing PACE without or with the split TEV protease treated with RAPA or ABA. Twenty-four hours after transfection, the HEK293T cells were treated with RAPA (**A**) or ABA (**B**), and 6 h later, viability was determined with propidium iodine staining.
(**C, D**) Inhibition of Ca^{2+}-dependent transcriptional activation with gadolinium chloride ($GdCl_3$). Twenty-four hours after transfection, the HEK293T cells were treated with $GdCl_3$ and RAPA (**C**) or ABA (**D**). Firefly and Renilla luciferase were measured 24 h later. The amounts of plasmids are listed in **Table S1**.
(**E, F**) Dual-luciferase assay at a concentration of 20 ng PACE. The bars (**A–F**) represent the mean ± s.d.; n = 4 biologically independent cell cultures.

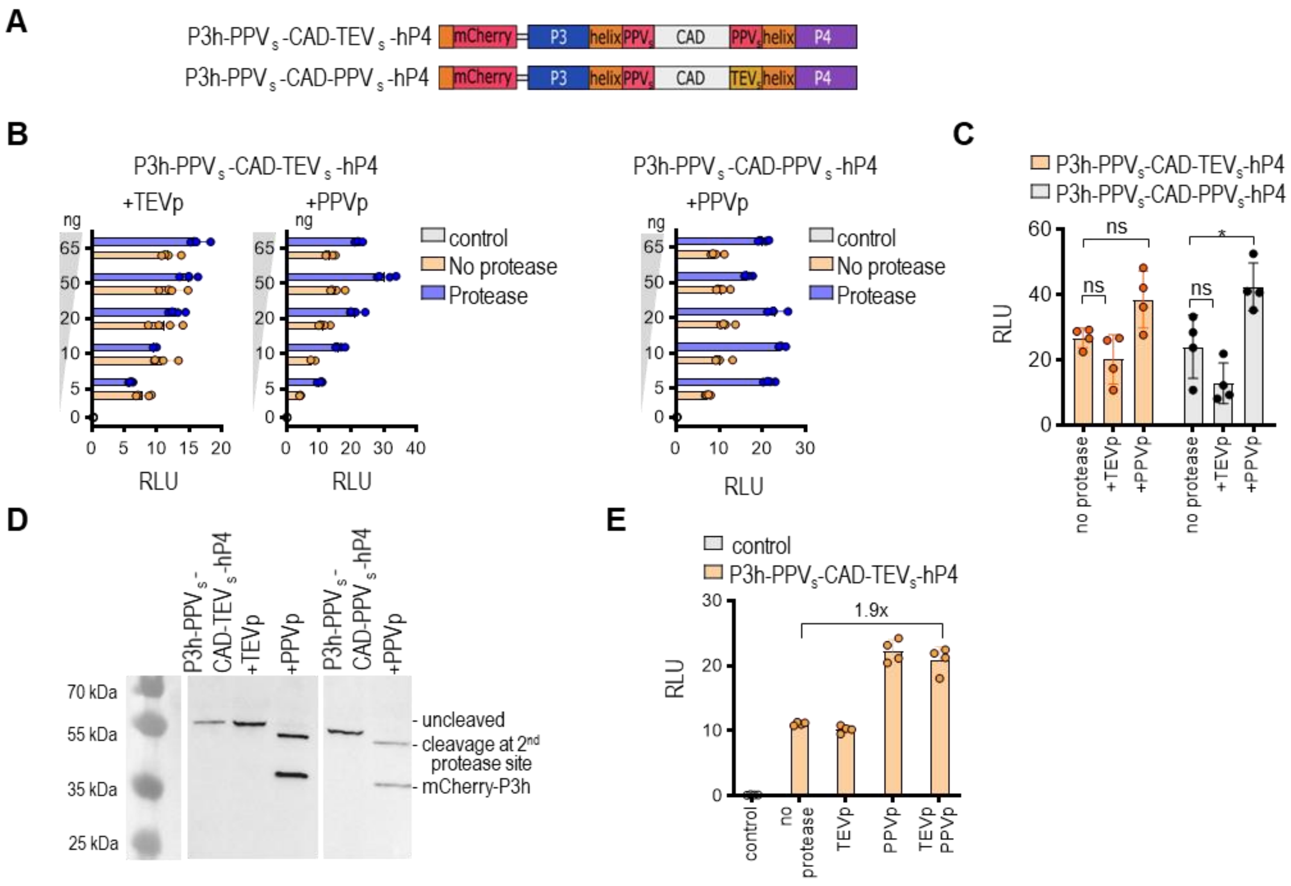

Fig. S7: PACE effector with dual regulation.

(**A**) PACE variants with various combinations of TEV_s and PPV_s protease recognition sites.

(**B, C, E**) Dual-luciferase assay of PACE variants in the presence of TEV_s and/or PPV_s. The amounts of transfected plasmids for all luciferase experiments are listed in **Table S1**. One day after transfection, the cells were lysed, and reporter activity was measured. The bars represent the mean ± s.d.; n = 4 biologically independent cell cultures. Statistical analyses and the corresponding p-values are listed in **Table S5**.

(**D**) Immunodetection of PACE without and in the presence of coexpressed TEV or PPV protease stained with anti-Myc antibodies.

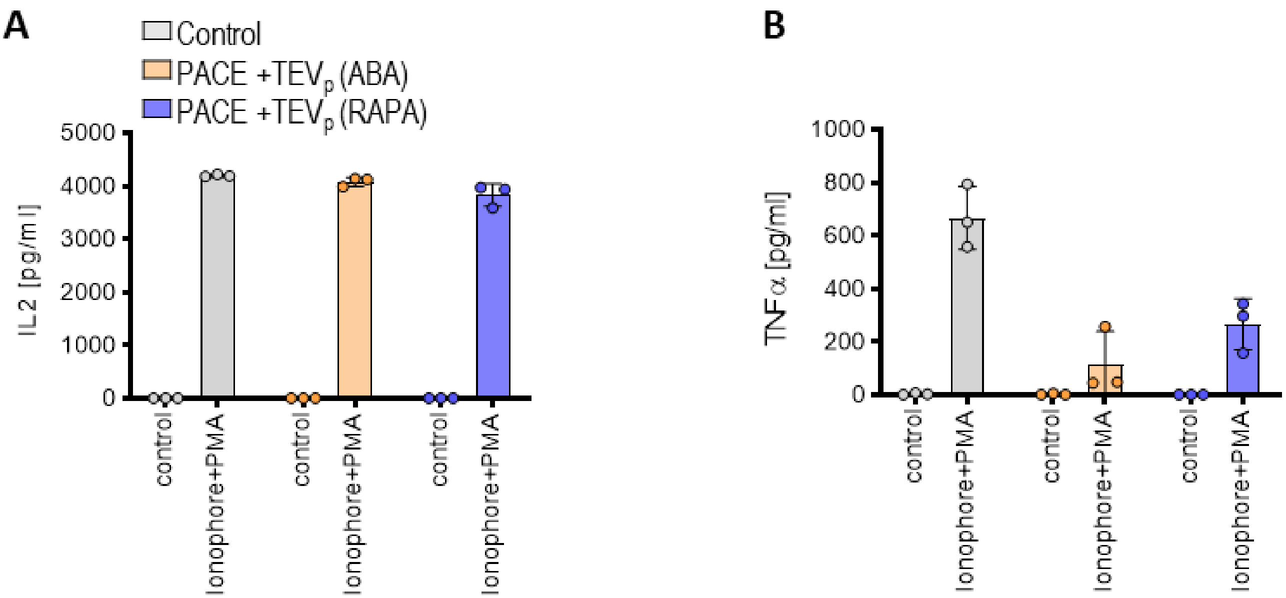

Fig. S8: ELISA assay for IL-2 (**A**) and TNFα (**B**) production in Jurkat T-cells expressing PACE and a split TEV protease. Production was induced with PMA and 2.5 µM ionophore. The amounts of transfected plasmids for all ELISA experiments are listed in **Table S4**. One day after transfection, the cells were centrifuged, and the supernatant was taken for ELISA.

SUPPLEMENTAL TABLES

Table S1: Amounts of transfected plasmids for HEK293T cells for each well in 96-well plates. Empty pcDNA3 backbone was used to equalize the amounts of transfected plasmids to 285 ng or 330 ng in case of figure 4H.

Input plasmid	Amount (ng)
**1C,S1**	
$_{10TALE}$_Pmin-fLuc<br>phRL-TK<br>NFAT-TALE-VP16-KRΦ<br>CAD	100<br>10<br>10<br>0, 0.5, 1, 2, 5, 10, 20, 35, 50, 65
**1D, 1E, 1F**	
$_{10TALE}$_Pmin-fLuc<br>phRL-TK<br>NFAT-TALE-VP16-KRΦ	100<br>10<br>10
P3-CAD or CAD-P4 or<br>P3-CAD-P4	0, 5, 10, 20, 50, 65
**1I**	
$_{10TALE}$_Pmin-fLuc<br>phRL-TK<br>NFAT-TALE-VP16-KRΦ<br>TEVp<br>P3-TEV$_s$-CAD-P4	100<br>10<br>10<br>50<br>0, 5, 10, 20, 50, 65
**2A,2B,2C,2D,2E,S2**	
$_{10TALE}$_Pmin-fLuc<br>phRL-TK<br>NFAT-TALE-VP16-KRΦ<br>TEVp	100<br>10<br>10<br>50
P3h-TEV$_s$-CAD-P4 or<br>P3-TEV$_s$-CAD-hP4 or<br>P3h-TEV$_s$-CAD-TEV$_s$-P4 or<br>P3-TEV$_s$-CAD-TEV$_s$-P4 or<br>PACE	0, 5, 10, 20, 50, 65
**3E, S6A, S6C, S6E**	
$_{10TALE}$_Pmin-fLuc<br>phRL-TK<br>NFAT-TALE-VP16-KRΦ<br>FRB-nTEVp<br>FKBP-cTEVp<br>PACE	100<br>10<br>10<br>50<br>50<br>0, 5, 10, 20, 50, 65
**3F, S6B, S6D, S6F**	
$_{10TALE}$_Pmin-fLuc<br>phRL-TK<br>NFAT-TALE-VP16-KRΦ<br>PYL1-nTEVp<br>ABI-cTEVp<br>PACE	100<br>10<br>10<br>50<br>50<br>0, 5, 10, 20, 50, 65
**4C, S7B**	
$_{10TALE}$_Pmin-fLuc<br>phRL-TK<br>NFAT-TALE-VP16-KRΦ	100<br>10<br>10
TEVp or<br>PPVp	50
P3h-PPV$_s$-CAD-TEV$_s$-hP4 or<br>P3h-TEV$_s$-CAD-PPV$_s$-hP4 or<br>P3h-PPV$_s$-CAD-PPV$_s$-hP4	0, 5, 10, 20, 50, 65
**4D,4f, S7C, S7E**	
$_{10TALE}$_Pmin-fLuc<br>phRL-TK<br>NFAT-TALE-VP16-KRΦ	100<br>10<br>10
TEVp or<br>PPVp or<br>both	50
P3h-PPV$_s$-CAD-TEV$_s$-hP4 or<br>P3h-TEV$_s$-CAD-PPV$_s$-hP4 or<br>P3h-PPV$_s$-CAD-PPV$_s$-hP4 or<br>PACE	20
**4H**	
$_{10TALE}$_Pmin-fLuc<br>phRL-TK<br>NFAT-TALE-VP16-KRΦ	50<br>10<br>10
PYL1-nTEVp<br>ABI-cTEVp<br>FRB-cPPVp<br>FKBP-nPPVp	50<br>50<br>50<br>50
P3h-TEV$_s$-CAD-PPV$_s$-hP4	0, 2, 5, 10, 20, 50

Table S2: Amounts of transfected plasmids for HEK293T cells for each well in 12-well plates used for Western blot analyses. Empty pcDNA3 backbone was used to equalize the amounts of transfected plasmids to 1,000 ng.

Input plasmid	Amount (ng)
**1G**	
P3-TEV$_s$-CAD-P4	500
TEVp	500
**2F, S3**	
P3-TEV$_s$-CAD-hP4 or<br>P3-TEV$_s$-CAD-hP4 or<br>PACE	500
TEVp	500
**4E, S7D**	
PACE or<br>P3h-PPV$_s$-CAD-TEV$_s$-hP4 or<br>P3h-TEV$_s$-CAD-PPV$_s$-hP4 or P3h-PPV$_s$-CAD-PPV$_s$-hP4	500
TEVp or PPVp	500

Table S3: Amounts of transfected plasmids for HEK293 cells for each well in eight-well plates used for microscopy. Empty pcDNA3 backbone was used to equalize the amounts of transfected plasmids to 250 ng.

Input plasmid	Amount (ng)
**1H, 2G upper**	
CAD or,<br>P3-TEV$_s$-CAC-P4 or<br>PACE	20
**2G lower**	
PACE<br>TEVp	20<br>50
**S4A**	
PACE<br>Orai	20<br>50
**S4B**	
PACE<br>Orai1<br>TEVp	20<br>50<br>50
**3C**	
FRB-nTEVp<br>FKBP-cTEVp<br>PACE	100<br>100<br>50
**3D**	
PYL1-nTEVp<br>ABI-cTEVp<br>PACE	100<br>100<br>50

Table S4: Amounts of electroporated plasmids for Jurkat cells for each 100 µl electroporation

Input plasmid	Amount (ng)
**5B**	
FRB-nTEVp<br>FKBP-cTEVp<br>PACE	3500<br>3500<br>3000
**5C**	
PYL1-nTEVp<br>ABI-cTEVp<br>PACE	3500<br>3500<br>3000

Table S5: Statistical analysis.

Figures	Test details	Significance	Summary	P value
**1C**	Unpaired t-test, two-tailed			
	0 vs 0,5 ng CAD	Yes	***	0,0005
	0 vs 1 ng CAD	Yes	***	<0,0001
	0 vs 2 ng CAD	Yes	***	0,0001
	0 vs 5 ng CAD	Yes	****	<0,0001
	0 vs 10 ng CAD	Yes	****	<0,0001
	0 vs 20 ng CAD	Yes	****	<0,0001
	0 vs 35 ng CAD	Yes	****	<0,0001
	0 vs 50 ng CAD	Yes	****	<0,0001
	0 vs 65 ng CAD	Yes	****	<0,0001
**1D**	Unpaired t-test, two-tailed			
	0 vs 5 ng P3-CAD	Yes	****	<0,0001
	0 vs 10 ng P3-CAD	Yes	****	<0,0001
	0 vs 20 ng P3-CAD	Yes	****	<0,0001
	0 vs 50 ng P3-CAD	Yes	****	<0,0001
	0 vs 65 ng P3-CAD	Yes	****	<0,0001
**1E**	Unpaired t-test, two-tailed			
	0 vs 5 ng CAD-P4	Yes	****	<0,0001
	0 vs 10 ng CAD-P4	Yes	****	<0,0001
	0 vs 20 ng CAD-P4	Yes	****	<0,0001
	0 vs 50 ng CAD-P4	Yes	****	<0,0001
	0 vs 65 ng CAD-P4	Yes	***	0,002
**1F**	One-way ANOVA<br>Tukey's multiple comparisons test			
	0 vs. 65 ng P3-CAD-P4	No	ns	0,4392
	0 vs. 20 ng CAD	Yes	****	<0,0001
	65 ng P3-CAD-P4 vs. 20 ng CAD	Yes	****	<0,0001
**1I**	One-way ANOVA<br>Dunnett's multiple comparisons test			
	0 vs. 5 ng P3-TEV$_s$-CAD-P4	No	ns	0,3428
	0 vs. 10 ng P3-TEV$_s$-CAD-P4	Yes	***	0,0006
	Unpaired t-test, two-tailed			
	5 ng P3-TEV$_s$-CAD-P4 +TEVp vs 5 ng P3-TEV$_s$-CAD-P4	Yes	*	0,0114
	10 ng P3-TEV$_s$-CAD-P4 +TEVp vs 10 ng P3-TEV$_s$-CAD-P4	Yes	*	0,0150
	20 ng P3-TEV$_s$-CAD-P4 +TEVp vs 20 ng P3-TEV$_s$-CAD-P4	Yes	**	0,0018
	50 ng P3-TEV$_s$-CAD-P4 +TEVp vs 50 ng P3-TEV$_s$-CAD-P4	Yes	****	<0,0001
	65 ng P3-TEV$_s$-CAD-P4 +TEVp vs 65 ng P3-TEV$_s$-CAD-P4	Yes	****	<0,0001
**2A**	One-way ANOVA<br>Dunnett's multiple comparisons test			
	0 vs. 5 ng P3h-TEV$_s$-CAD-P4	No	ns	0,8716
	0 vs. 10 ng P3h-TEV$_s$-CAD-P4	No	ns	0,1845
	0 vs. 20 ng P3h-TEV$_s$-CAD-P4	No	ns	0,1014
	0 vs. 50 ng P3h-TEV$_s$-CAD-P4	Yes	*	0,0298
	0 vs. 65 ng P3h-TEV$_s$-CAD-P4	Yes	**	0,0094
	Unpaired t-test, two-tailed			
	5 ng P3h-TEV$_s$-CAD-P4 vs 5 ng P3h-TEV$_s$-CAD-P4 +TEVp	Yes	**	0,0014
	10 ng P3h-TEV$_s$-CAD-P4 vs 10 ng P3h-TEV$_s$-CAD-P4 +TEVp	Yes	*	0,0188
	20 ng P3h-TEV$_s$-CAD-P4 vs 20 ng P3h-TEV$_s$-CAD-P4 +TEVp	Yes	ns	0,0527
	50 ng P3h-TEV$_s$-CAD-P4 vs 50 ng P3h-TEV$_s$-CAD-P4 +TEVp	No	ns	0,6138
	65 ng P3h-TEV$_s$-CAD-P4 vs 65 ng P3h-TEV$_s$-CAD-P4 +TEVp	Yes	ns	0,0912
**2B**	One-way ANOVA<br>Dunnett's multiple comparisons test			
	0 vs. 5 ng P3-TEV$_s$-CAD-hP4	No	ns	0,2180
	0 vs. 10 ng P3-TEV$_s$-CAD-hP4	Yes	***	0,0006
	0 vs. 20 ng P3-TEV$_s$-CAD-hP4	Yes	****	<0,0001
	0 vs. 50 ng P3-TEV$_s$-CAD-hP4	Yes	****	<0,0001
	0 vs. 65 ng P3-TEV$_s$-CAD-hP4	Yes	****	<0,0001
	Unpaired t-test, two-tailed			
	5 ng P3-TEV$_s$-CAD-hP4 +TEVp vs 5 ng P3-TEV$_s$-CAD-hP4	Yes	***	0,0004
	10 ng P3-TEV$_s$-CAD-hP4 +TEVp vs 10 ng P3-TEV$_s$-CAD-hP4	Yes	***	0,0003
	20 ng P3-TEV$_s$-CAD-hP4 +TEVp vs 20 ng P3-TEV$_s$-CAD-hP4	Yes	***	0,0003
	50 ng P3-TEV$_s$-CAD-hP4 +TEVp vs 50 ng P3-TEV$_s$-CAD-hP4	Yes	**	0,0024
	65 ng P3-TEV$_s$-CAD-hP4 +TEVp vs 65 ng P3-TEV$_s$-CAD-hP4	Yes	*	0,0216
**2C**	One-way ANOVA<br>Dunnett's multiple comparisons test			
	0 vs. 5 ng P3h-TEV$_s$-CAD-TEV-P4	No	ns	0,9568
	0 vs. 10 ng P3h-TEV$_s$-CAD--TEVhPV	No	ns	0,3283
	0 vs. 20 ng P3h-TEV$_s$-CAD-TEV-P4	Yes	*	0,0386
	0 vs. 50 ng P3h-TEV$_s$-CAD-TEV-P4	Yes	****	<0,0001
	0 vs. 65 ng P3h-TEV$_s$-CAD-TEV-P4	Yes	****	<0,0001
	Unpaired t-test, two-tailed			
	5 ng P3h-TEV$_s$-CAD-TEV$_s$-P4 vs 5 ng P3h-TEV$_s$-CAD-TEV$_s$-P4 +TEVp	Yes	****	<0,0001
	10 ng P3h-TEV$_s$-CAD-TEV$_s$-P4 vs 10 ng P3h-TEV$_s$-CAD-TEV$_s$-P4 +TEVp	Yes	****	<0,0001
	20 ng P3h-TEV$_s$-CAD-TEV$_s$-P4 vs 20 ng P3h-TEV$_s$-CAD-TEV$_s$-P4 +TEVp	Yes	****	<0,0001
	50 ng P3h-TEV$_s$-CAD-TEV$_s$-P4 vs 50 ng P3h-TEV$_s$-CAD-TEV$_s$-P4 +TEVp	Yes	****	<0,0001
	65 ng P3h-TEV$_s$-CAD-TEV$_s$-P4 vs 65 ng P3h-TEV$_s$-CAD-TEV$_s$-P4 +TEVp	Yes	****	<0,0001

**2D**	One-way ANOVA			
	Dunnett's multiple comparisons test			
	0 vs. 5 ng P3-TEV$_s$-CAD-TEV$_s$-hP4	Yes	*	0,0208
	0 vs. 10 ng P3-TEV$_s$-CAD-TEV$_s$-hP4	Yes	****	<0,0001
	0 vs. 20 ng P3-TEV$_s$-CAD-TEV$_s$-hP4	Yes	****	<0,0001
	0 vs. 50 ng P3-TEV$_s$-CAD-TEV$_s$-hP4	Yes	****	<0,0001
	0 vs. 65 ng P3-TEV$_s$-CAD-TEV$_s$-hP4	Yes	****	<0,0001
	Unpaired t-test, two-tailed			
	5 ng P3-TEV$_s$-CAD-TEV$_s$-hP4 vs 5 ng P3-TEV$_s$-CAD-hTEV$_s$-P4 +TEVp	Yes	****	<0,0001
	10 ng P3-TEV$_s$-CAD-TEV$_s$-hP4 vs 10 ng P3-TEV$_s$-CAD-hTEV$_s$-P4 +TEVp	Yes	****	<0,0001
	20 ng P3-TEV$_s$-CAD-TEV$_s$-hP4 vs 20 ng P3-TEV$_s$-CAD-hTEV$_s$-P4 +TEVp	Yes	****	<0,0001
	50 ng P3-TEV$_s$-CAD-TEV$_s$-hP4 vs 50 ng P3-TEV$_s$-CAD-hTEV$_s$-P4 +TEVp	Yes	****	<0,0001
	65 ng P3-TEV$_s$-CAD-TEV$_s$-hP4 vs 65 ng P3-TEV$_s$-CAD-hTEV$_s$-P4 +TEVp	Yes	****	<0,0001
**2E**	One-way ANOVA			
	Dunnett's multiple comparisons test			
	0 vs. 5 ng P3h-TEV$_s$-CAD-TEV$_s$-hP4	Yes	**	0,0020
	0 vs. 10 ng P3h-TEV$_s$-CAD-TEV$_s$-hP4	Yes	****	<0,0001
	0 vs. 20 ng P3h-TEV$_s$-CAD-TEV$_s$-hP4	Yes	****	<0,0001
	0 vs. 50 ng P3h-TEV$_s$-CAD-TEV$_s$-hP4	No	ns	0,5747
	0 vs. 65 ng P3h-TEV$_s$-CAD-TEV$_s$-hP4	No	ns	0,3845
	Unpaired t-test, two-tailed			
	5 ng P3h-TEV$_s$-CAD-TEV$_s$-hP4 vs 5 ng P3h-TEV$_s$-CAD-hTEV$_s$-P4 +TEVp	Yes	****	<0,0001
	10 ng P3h-TEV$_s$-CAD-TEV$_s$-hP4 vs 10 ng P3h-TEV$_s$-CAD-hTEV$_s$-P4 +TEVp	Yes	****	<0,0001
	20 ng P3h-TEV$_s$-CAD-TEV$_s$-hP4 vs 20 ng P3h-TEV$_s$-CAD-hTEV$_s$-P4 +TEVp	Yes	****	<0,0001
	50 ng P3h-TEV$_s$-CAD-TEV$_s$-hP4 vs 50 ng P3h-TEV$_s$-CAD-hTEV$_s$-P4 +TEVp	Yes	****	<0,0001
	65 ng P3h-TEV$_s$-CAD-TEV$_s$-hP4 vs 65 ng P3h-TEV$_s$-CAD-hTEV$_s$-P4 +TEVp	Yes	****	<0,0001
**3E**	One-way ANOVA			
	Dunnett's multiple comparisons test			
	0 vs. 0+RAPA	No	ns	0,9997
	0 vs. 5 ng PACE	No	ns	0,9993
	0 vs. 10 ng PACE	No	ns	0,3138
	0 vs. 20 ng PACE	No	ns	0,9438
	0 vs. 50 ng PACE	No	ns	0,7392
	0 vs. 65 ng PACE	No	ns	0,8008
	Unpaired t-test, two-tailed			
	5 ng PACE +RAPA vs, 5 ng PACE	Yes	****	<0,0001
	10 ng PACE +RAPA vs, 10 ng PACE	Yes	***	0,0004
	20 ng PACE +RAPA vs, 20 ng PACE	Yes	****	<0,0001
	50 ng PACE +RAPA vs, 50 ng PACE	Yes	****	<0,0001
	65 ng PACE +RAPA vs, 65 ng PACE	Yes	****	<0,0001
**3F**	One-way ANOVA			
	Dunnett's multiple comparisons test			
	0 vs. 0+ABA	No	ns	0,9996
	0 vs. 5 ng PACE	Yes	****	<0,0001
	0 vs. 10 ng PACE	Yes	***	0,0004
	0 vs. 20 ng PACE	Yes	****	<0,0001
	0 vs. 50 ng PACE	Yes	***	0,0008
	0 vs. 65 ng PACE	Yes	****	<0,0001
	Unpaired t-test, two-tailed			
	5 ng PACE + ABA vs, 5 ng PACE	No	Ns	0,1490
	10 ng PACE +ABA vs, 10 ng PACE	Yes	****	<0,0001
	20 ng PACE +ABA vs, 20 ng PACE	Yes	****	<0,0001
	50 ng PACE +ABA vs, 50 ng PACE	Yes	****	<0,0001
	65 ng PACE +ABA vs, 65 ng PACE	Yes	****	<0,0001
**4C 1**	One-way ANOVA			
	Dunnett's multiple comparisons test			
	0 vs. 5 ng P3-TEV$_s$-CAD-PPV$_s$-hP4	Yes	****	<0,0001
	0 vs. 10 ng P3-TEV$_s$-CAD-PPV$_s$-hP4	Yes	****	<0,0001
	0 vs. 20 ng P3-TEV$_s$-CAD-PPV$_s$-hP4	Yes	****	<0,0001
	0 vs. 50 ng P3-TEV$_s$-CAD-PPV$_s$-hP4	Yes	****	<0,0001
	0 vs. 65 ng P3-TEV$_s$-CAD-PPV$_s$-hP4	Yes	****	<0,0001
	Unpaired t-test, two-tailed			
	5 ng P3-TEV$_s$-CAD-PPV$_s$-hP4 +TEVp vs 5 ng P3-TEV$_s$-CAD-PPV$_s$-hP4	Yes	*	0,0182
	10 ng P3-TEV$_s$-CAD-PPV$_s$-hP4 +TEVp vs 10 ng P3-TEV$_s$-CAD-PPV$_s$-hP4	No	ns	0,2241
	20 ng P3-TEV$_s$-CAD-PPV$_s$-hP4 +TEVp vs 20 ng P3-TEV$_s$-CAD-PPV$_s$-hP4	No	ns	0,2384
	50 ng P3-TEV$_s$-CAD-PPV$_s$-hP4 +TEVp vs 50 ng P3-TEV$_s$-CAD-PPV$_s$-hP4	No	ns	0,0543
	65 ng P3-TEV$_s$-CAD-PPV$_s$-hP4 +TEVp vs 65 ng P3-TEV$_s$-CAD-PPV$_s$-hP4	Yes	**	0,0033
**4C 2**	One-way ANOVA			
	Dunnett's multiple comparisons test			
	0 vs. 5 ng P3-TEV$_s$-CAD-PPV$_s$-hP4	Yes	**	0,0050
	0 vs. 10 ng P3-TEV$_s$-CAD-PPV$_s$-hP4	Yes	****	<0,0001
	0 vs. 20 ng P3-TEV$_s$-CAD-PPV$_s$-hP4	Yes	****	<0,0001
	0 vs. 50 ng P3-TEV$_s$-CAD-PPV$_s$-hP4	Yes	****	<0,0001
	0 vs. 65 ng P3-TEV$_s$-CAD-PPV$_s$-hP4	Yes	****	<0,0001
	Unpaired t-test, two-tailed			
	5 ng P3-TEV$_s$-CAD-PPV$_s$-hP4 +PPVp vs 5 ng P3-TEV$_s$-CAD-PPV$_s$-hP4	Yes	****	<0,0001
	10 ng P3-TEV$_s$-CAD-PPV$_s$-hP4 +PPVp vs 10 ng P3-TEV$_s$-CAD-PPV$_s$-hP4	Yes	****	<0,0001
	20 ng P3-TEV$_s$-CAD-PPV$_s$-hP4 +PPVp vs 20 ng P3-TEV$_s$-CAD-PPV$_s$-hP4	Yes	***	0,0003
	50 ng P3-TEV$_s$-CAD-PPV$_s$-hP4 +PPVp vs 50 ng P3-TEV$_s$-CAD-PPV$_s$-hP4	Yes	***	0,0001

	65 ng P3-TEV$_s$-CAD-PPV$_s$-hP4 +PPVp vs 65 ng P3-TEV$_s$-CAD-PPV$_s$-hP4	Yes	****	<0,0001
**4D**	One-way ANOVA<br>Dunnett's multiple comparisons test			
	PACE vs. PACE +TEVp	Yes	****	<0,0001
	PACE vs. PACE +PPVp	No	ns	0,6673
	P3h-TEV$_s$-CAD-PPV-hP4 vs. P3h-TEV$_s$-CAD-PPV$_s$-hP4 +TEVp	Yes	**	0,0063
	P3h-TEV$_s$-CAD-PPV-hP4 vs. P3h-TEV$_s$-CAD-PPV$_s$-hP4 +PPVp	Yes	****	<0,0001
**4F**	One-way ANOVA<br>Dunnett's multiple comparisons test			
	P3h-TEV$_s$-CAD-PPV$_s$-hP4 vs 0	No	ns	0,475
	P3h-TEV$_s$-CAD-PPV$_s$-hP4 vs. P3h-TEV$_s$-CAD-PPV$_s$-hP4 +TEVp	Yes	****	<0,0001
	P3h-TEV$_s$-CAD-PPV$_s$-hP4 vs. P3h-TEV$_s$-CAD-PPV$_s$-hP4 +PPVp	Yes	****	<0,0001
	P3h-TEV$_s$-CAD-PPV$_s$-hP4 vs. P3h-TEV$_s$-CAD-PPV$_s$-hP4 +TEVp +PPVp	Yes	****	<0,0001
**4H**	One-way ANOVA<br>Dunnett's multiple comparisons test			
	20 ng PACE-OR vs. 20 ng PACE-OR RAPA	Yes	****	<0,0001
	20 ng PACE-OR vs. 20 ng PACE-OR ABA	Yes	***	0,0002
	20 ng PACE-OR vs. 20 ng PACE-OR RAPA + ABA	Yes	****	<0,0001
**5B**	One-way ANOVA<br>Dunnett's multiple comparisons test			
	Control vs. Control +PMA	No	ns	0,1494
	Control vs. Control +PMA +ABA	No	ns	0,1691
	Control vs. Control +PMA +RAPA	No	ns	0,1912
	PACE+TEVp(ABA) vs. PACE+TEVp(ABA) +PMA	Yes	*	0,0498
	PACE+TEVp(ABA) vs. PACE+TEVp(ABA) +PMA +ABA	Yes	****	<0,0001
	PACE+TEVp(ABA) vs. PACE+TEVp(ABA) +PMA	No	ns	0,0619
	PACE+TEVp(ABA) vs. PACE+TEVp(ABA) +PMA +ABA	Yes	****	<0,0001
**5C**	One-way ANOVA<br>Dunnett's multiple comparisons test			
	Control vs. Control +PMA	No	ns	0,9553
	Control vs. Control +PMA +ABA	No	ns	0,9465
	Control vs. Control +PMA +RAPA	No	ns	0,7775
	PACE+TEVp(ABA) vs. PACE+TEVp(ABA) +PMA	No	ns	0,8132
	PACE+TEVp(ABA) vs. PACE+TEVp(ABA) +PMA +ABA	Yes	**	0,0048
	PACE+TEVp(ABA) vs. PACE+TEVp(ABA) +PMA	Yes	*	0,0292
	PACE+TEVp(ABA) vs. PACE+TEVp(ABA) +PMA +ABA	No	ns	0,0855
**S7B 1**	One-way ANOVA<br>Dunnett's multiple comparisons test			
	0 vs. 5 ng P3-PPV$_s$-CAD-TEV$_s$-hP4	No	ns	0,8742
	0 vs. 10 ng P3-PPV$_s$-CAD-TEV$_s$-hP4	No	ns	0,4414
	0 vs. 20 ng P3-PPV$_s$-CAD-TEV$_s$-hP4	No	ns	0,0641
	0 vs. 50 ng P3-PPV$_s$-CAD-TEV$_s$-hP4	No	ns	0,0989
	0 vs. 65 ng P3-PPV$_s$-CAD-TEV$_s$-hP4	No	ns	0,2220
	Unpaired t-test, two-tailed			
	5 ng P3-PPV$_s$-CAD-TEV$_s$-hP4 +TEVp vs 5 ng P3-PPV$_s$-CAD-TEV$_s$-hP4	Yes	****	<0,0001
	10 ng P3-PPV$_s$-CAD-TEV$_s$-hP4 +TEVp vs 10 ng P3-PPV$_s$-CAD-TEV$_s$-hP4	Yes	****	<0,0001
	20 ng P3-PPV$_s$-CAD-TEV$_s$-hP4 +TEVp vs 20 ng P3-PPV$_s$-CAD-TEV$_s$-hP4	Yes	****	<0,0001
	50 ng P3-PPV$_s$-CAD-TEV$_s$-hP4 +TEVp vs 50 ng P3-PPV$_s$-CAD-TEV$_s$-hP4	Yes	****	<0,0001
	65 ng P3-PPV$_s$-CAD-TEV$_s$-hP4 +TEVp vs 65 ng P3-PPV$_s$-CAD-TEV$_s$-hP4	Yes	****	<0,0001
**S7B 2**	One-way ANOVA<br>Dunnett's multiple comparisons test			
	0 vs. 5 ng P3-PPV$_s$-CAD-TEV$_s$-hP4	No	****	<0,0001
	0 vs. 10 ng P3-PPV$_s$-CAD-TEV$_s$-hP4	No	****	<0,0001
	0 vs. 20 ng P3-PPV$_s$-CAD-TEV$_s$-hP4	Yes	****	<0,0001
	0 vs. 50 ng P3-PPV$_s$-CAD-TEV$_s$-hP4	Yes	****	<0,0001
	0 vs. 65 ng P3-PPV$_s$-CAD-TEV$_s$-hP4	Yes	****	<0,0001
	Unpaired t-test, two-tailed			
	5 ng P3-PPV$_s$-CAD-TEV$_s$-hP4 +PPVp vs 5 ng P3-PPV$_s$-CAD-TEV$_s$-hP4	Yes	ns	0,6462
	10 ng P3-PPV$_s$-CAD-TEV$_s$-hP4 +PPVp vs 10 ng P3-PPV$_s$-CAD-TEV$_s$-hP4	Yes	ns	0,2593
	20 ng P3-PPV$_s$-CAD-TEV$_s$-hP4 +PPVp vs 20 ng P3-PPV$_s$-CAD-TEV$_s$-hP4	Yes	*	0,0325
	50 ng P3-PPV$_s$-CAD-TEV$_s$-hP4 +PPVp vs 50 ng P3-PPV$_s$-CAD-TEV$_s$-hP4	Yes	*	0,0128
	65 ng P3-PPV$_s$-CAD-TEV$_s$-hP4 +PPVp vs 65 ng P3-PPV$_s$-CAD-TEV$_s$-hP4	Yes	**	0,0064
**S7B 3**	One-way ANOVA<br>Dunnett's multiple comparisons test			
	0 vs. 5 ng P3-PPV$_s$-CAD-PPV$_s$-hP4	Yes	****	<0,0001
	0 vs. 10 ng P3-PPV$_s$-CAD-PPV$_s$-hP4	Yes	****	<0,0001
	0 vs. 20 ng P3-PPV$_s$-CAD-PPV$_s$-hP4	Yes	****	<0,0001
	0 vs. 50 ng P3-PPV$_s$-CAD-PPV$_s$-hP4	Yes	****	<0,0001
	0 vs. 65 ng P3-PPV$_s$-CAD-PPV$_s$-hP4	Yes	****	<0,0001
	Unpaired t-test, two-tailed			
	5 ng P3-PPV$_s$-CAD-PPV$_s$-hP4 +PPVp vs 5 ng P3-PPV$_s$-CAD-PPV$_s$-hP4	Yes	****	<0,0001
	10 ng P3-PPV$_s$-CAD-PPV$_s$-hP4 +PPVp vs 10 ng P3-PPV$_s$-CAD-PPV$_s$-hP4	Yes	****	<0,0001
	20 ng P3-PPV$_s$-CAD-PPV$_s$-hP4 +PPVp vs 20 ng P3-PPV$_s$-CAD-PPV$_s$-hP4	Yes	****	<0,0001
	50 ng P3-PPV$_s$-CAD-PPV$_s$-hP4 +PPVp vs 50 ng P3-PPV$_s$-CAD-PPV$_s$-hP4	Yes	***	0,0004
	65 ng P3-PPV$_s$-CAD-PPV$_s$-hP4 +PPVp vs 65 ng P3-PPV$_s$-CAD-PPV$_s$-hP4	Yes	****	<0,0001
**S7C**	One-way ANOVA<br>Dunnett's multiple comparisons test			
	P3h-PPV$_s$-CAD-TEV$_s$-hP4 vs. P3h-PPV$_s$-CAD-TEV$_s$-hP4 +TEVp	No	ns	0,3455

	P3h-PPV$_s$-CAD-TEV$_s$-hP4 vs. P3h-PPV$_s$-CAD-TEV$_s$-hP4 +PPVp	No	ns	0,0698
	P3h-PPV$_s$-CAD-PPV$_s$-hP4 vs. P3h-PPV$_s$-CAD-PPV$_s$-hP4 +TEVp	No	ns	0,1309
	P3h-PPV$_s$-CAD-PPV$_s$-hP4 vs. P3h-PPV$_s$-CAD-PPV$_s$-hP4 +PPVp	Yes	*	0,0169
**S7E**	One-way ANOVA<br>Dunnett's multiple comparisons test			
	P3h-PPV$_s$-CAD-TEV$_s$-hP4 vs 0	Yes	****	<0,0001
	P3h-PPV$_s$-CAD-TEV$_s$-hP4 vs. P3h-PPV$_s$-CAD-TEV$_s$-hP4 +TEVp	No	ns	0,7259
	P3h-PPV$_s$-CAD-TEV$_s$-hP4 vs. P3h-PPV$_s$-CAD-TEV$_s$-hP4 +PPVp	Yes	****	<0,0001
	P3h-PPV$_s$-CAD-TEV$_s$-hP4 vs. P3h-PPV$_s$-CAD-TEV$_s$-hP4 +TEVp +PPVp	Yes	****	<0,0001

Table S6: List of constructs with the amino acid sequences used in this study

	Construct name	Color coding
1	CAD	Myc, mCherry, P3, alpha helix, TEVp cleavage site, CAD, P4, PPVp cleavage site

>mCherry
MVSKGEEDNMAIIKEFMRFKVHMEGSVNGHEFEIEGEGEGRPYEGTQTAKLKVTKGGPLPFAWDILSPQFMYGSKAYVKHPADIPDYLKLSFPE
GFKWERVMNFEDGGVVTVTQDSSLQDGEFIYKVKLRGTNFPSDGPVMQKKTMGWEASSERMYPEDGALKGEIKQRLKLKDGGHYDAEVKTT
YKAKKPVQLPGAYNVNIKLDITSHNEDYTIVEQYERAEGRHSTGGMDELYKSGLRSRAQASNSYAPE
>CAD
ALQKWLQLTHEVEVQYYNIKKQNAEKQLLVAKEGAEKIKKKRNTLFGTFHVAHSSSLDDVDHKILTAKQALSEVTAALRERLHRWQQIEILCGF
QIVNNPGIH

2	P3-CAD	

>mCherry
MVSKGEEDNMAIIKEFMRFKVHMEGSVNGHEFEIEGEGEGRPYEGTQTAKLKVTKGGPLPFAWDILSPQFMYGSKAYVKHPADIPDYLKLSFPE
GFKWERVMNFEDGGVVTVTQDSSLQDGEFIYKVKLRGTNFPSDGPVMQKKTMGWEASSERMYPEDGALKGEIKQRLKLKDGGHYDAEVKTT
YKAKKPVQLPGAYNVNIKLDITSHNEDYTIVEQYERAEGRHSTGGMDELYKSGLRSRAQASNSYAPE
>P3
IQQLEEEIAQLEQKNAALKEKNQALKYG
>CAD
ALQKWLQLTHEVEVQYYNIKKQNAEKQLLVAKEGAEKIKKKRNTLFGTFHVAHSSSLDDVDHKILTAKQALSEVTAALRERLHRWQQIEILCGF
QIVNNPGIH

3	CAD-P4	

>mCherry
MVSKGEEDNMAIIKEFMRFKVHMEGSVNGHEFEIEGEGEGRPYEGTQTAKLKVTKGGPLPFAWDILSPQFMYGSKAYVKHPADIPDYLKLSFPE
GFKWERVMNFEDGGVVTVTQDSSLQDGEFIYKVKLRGTNFPSDGPVMQKKTMGWEASSERMYPEDGALKGEIKQRLKLKDGGHYDAEVKTT
YKAKKPVQLPGAYNVNIKLDITSHNEDYTIVEQYERAEGRHSTGGMDELYKSGLRSRAQASNSYAPE
>CAD
ALQKWLQLTHEVEVQYYNIKKQNAEKQLLVAKEGAEKIKKKRNTLFGTFHVAHSSSLDDVDHKILTAKQALSEVTAALRERLHRWQQIEILCGF
QIVNNPGIH
>P4
KIAQLKQKIQALKQENQQLEEENAALEYG

4	P3-CAD	

>mCherry
MVSKGEEDNMAIIKEFMRFKVHMEGSVNGHEFEIEGEGEGRPYEGTQTAKLKVTKGGPLPFAWDILSPQFMYGSKAYVKHPADIPDYLKLSFPE
GFKWERVMNFEDGGVVTVTQDSSLQDGEFIYKVKLRGTNFPSDGPVMQKKTMGWEASSERMYPEDGALKGEIKQRLKLKDGGHYDAEVKTT
YKAKKPVQLPGAYNVNIKLDITSHNEDYTIVEQYERAEGRHSTGGMDELYKSGLRSRAQASNSYAPE
>P3
IQQLEEEIAQLEQKNAALKEKNQALKYG
>TEV cleavage site
ENLYFQS
>CAD
ALQKWLQLTHEVEVQYYNIKKQNAEKQLLVAKEGAEKIKKKRNTLFGTFHVAHSSSLDDVDHKILTAKQALSEVTAALRERLHRWQQIEILCGF
QIVNNPGIH

5	P3-CAD-P4	

>mCherry
MVSKGEEDNMAIIKEFMRFKVHMEGSVNGHEFEIEGEGEGRPYEGTQTAKLKVTKGGPLPFAWDILSPQFMYGSKAYVKHPADIPDYLKLSFPE
GFKWERVMNFEDGGVVTVTQDSSLQDGEFIYKVKLRGTNFPSDGPVMQKKTMGWEASSERMYPEDGALKGEIKQRLKLKDGGHYDAEVKTT
YKAKKPVQLPGAYNVNIKLDITSHNEDYTIVEQYERAEGRHSTGGMDELYKSGLRSRAQASNSYAPE
>P3
IQQLEEEIAQLEQKNAALKEKNQALKYG
>TEV cleavage site
ENLYFQS
>CAD
ALQKWLQLTHEVEVQYYNIKKQNAEKQLLVAKEGAEKIKKKRNTLFGTFHVAHSSSLDDVDHKILTAKQALSEVTAALRERLHRWQQIEILCGF
QIVNNPGIH
>P4
KIAQLKQKIQALKQENQQLEEENAALEYG

6	P3-TEV$_s$-CAD-P4	

>mCherry
MVSKGEEDNMAIIKEFMRFKVHMEGSVNGHEFEIEGEGEGRPYEGTQTAKLKVTKGGPLPFAWDILSPQFMYGSKAYVKHPADIPDYLKLSFPE
GFKWERVMNFEDGGVVTVTQDSSLQDGEFIYKVKLRGTNFPSDGPVMQKKTMGWEASSERMYPEDGALKGEIKQRLKLKDGGHYDAEVKTT
YKAKKPVQLPGAYNVNIKLDITSHNEDYTIVEQYERAEGRHSTGGMDELYKSGLRSRAQASNSYAPE
>P3
IQQLEEEIAQLEQKNAALKEKNQALKYG
>TEV cleavage site
ENLYFQS
>CAD
ALQKWLQLTHEVEVQYYNIKKQNAEKQLLVAKEGAEKIKKKRNTLFGTFHVAHSSSLDDVDHKILTAKQALSEVTAALRERLHRWQQIEILCGF
QIVNNPGIH
>P4
KIAQLKQKIQALKQENQQLEEENAALEYG

7	P3-TEV$_s$-CAD-hP4	

>myc
MEQKLISEEDL
>mCherry
VSKGEEDNMAIIKEFMRFKVHMEGSVNGHEFEIEGEGEGRPYEGTQTAKLKVTKGGPLPFAWDILSPQFMYGSKAYVKHPADIPDYLKLSFPEGF
KWERVMNFEDGGVVTVTQDSSLQDGEFIYKVKLRGTNFPSDGPVMQKKTMGWEASSERMYPEDGALKGEIKQRLKLKDGGHYDAEVKTTYK
AKKPVQLPGAYNVNIKLDITSHNEDYTIVEQYERAEGRHSTGGMDELYKSGLRSRAQASNSYAPE
>P3
IQQLEEEIAQLEQKNAALKEKNQALKYG
>TEV cleavage site
ENLYFQS
>CAD
ALQKWLQLTHEVEVQYYNIKKQNAEKQLLVAKEGAEKIKKKRNTLFGTFHVAHSSSLDDVDHKILTAKQALSEVTAALRERLHRWQQIEILCGF
QIVNNPGIH
>P4
EEEEKKKKEEEEKKKIAQLKQKIQALKQENQQLEEENAALEYG

8	P3h-TEV$_s$-CAD-P4	

>myc
MEQKLISEEDL
>mCherry
VSKGEEDNMAIIKEFMRFKVHMEGSVNGHEFEIEGEGEGRPYEGTQTAKLKVTKGGPLPFAWDILSPQFMYGSKAYVKHPADIPDYLKLSFPEGF
KWERVMNFEDGGVVTVTQDSSLQDGEFIYKVKLRGTNFPSDGPVMQKKTMGWEASSERMYPEDGALKGEIKQRLKLKDGGHYDAEVKTTYK
AKKPVQLPGAYNVNIKLDITSHNEDYTIVEQYERAEGRHSTGGMDELYKSGLRSRAQASNSYAPE
>P3
IQQLEEEIAQLEQKNAALKEKNQALKYEEEEKKKKEEEEKKG
>TEV cleavage site
ENLYFQS
>CAD
ALQKWLQLTHEVEVQYYNIKKQNAEKQLLVAKEGAEKIKKKRNTLFGTFHVAHSSSLDDVDHKILTAKQALSEVTAALRERLHRWQQIEILCGF
QIVNNPGIH
>P4
KIAQLKQKIQALKQENQQLEEENAALEYG

9	P3-TEV$_s$-CAD-TEV$_s$-hP4	

>myc
MEQKLISEEDL
>mCherry
VSKGEEDNMAIIKEFMRFKVHMEGSVNGHEFEIEGEGEGRPYEGTQTAKLKVTKGGPLPFAWDILSPQFMYGSKAYVKHPADIPDYLKLSFPEGF
KWERVMNFEDGGVVTVTQDSSLQDGEFIYKVKLRGTNFPSDGPVMQKKTMGWEASSERMYPEDGALKGEIKQRLKLKDGGHYDAEVKTTYK
AKKPVQLPGAYNVNIKLDITSHNEDYTIVEQYERAEGRHSTGGMDELYKSGLRSRAQASNSYAPE
>P3
IQQLEEEIAQLEQKNAALKEKNQALKYEEEEKKKKEEEEKKG
>TEV cleavage site
ENLYFQS
>CAD
ALQKWLQLTHEVEVQYYNIKKQNAEKQLLVAKEGAEKIKKKRNTLFGTFHVAHSSSLDDVDHKILTAKQALSEVTAALRERLHRWQQIEILCGF
QIVNNPGIH
>TEV cleavage site
GPGENLYFQSGSG
>P4
EEEEKKKKEEEEKKKIAQLKQKIQALKQENQQLEEENAALEYG

10	P3h-TEV$_s$-CAD-TEV$_s$-P4	

>myc
MEQKLISEEDL
>mCherry
VSKGEEDNMAIIKEFMRFKVHMEGSVNGHEFEIEGEGEGRPYEGTQTAKLKVTKGGPLPFAWDILSPQFMYGSKAYVKHPADIPDYLKLSFPEGF
KWERVMNFEDGGVVTVTQDSSLQDGEFIYKVKLRGTNFPSDGPVMQKKTMGWEASSERMYPEDGALKGEIKQRLKLKDGGHYDAEVKTTYK
AKKPVQLPGAYNVNIKLDITSHNEDYTIVEQYERAEGRHSTGGMDELYKSGLRSRAQASNSYAPE
>P3
IQQLEEEIAQLEQKNAALKEKNQALKYEEEEKKKKEEEEKKG
>TEV cleavage site
ENLYFQS
>CAD
ALQKWLQLTHEVEVQYYNIKKQNAEKQLLVAKEGAEKIKKKRNTLFGTFHVAHSSSLDDVDHKILTAKQALSEVTAALRERLHRWQQIEILCGF
QIVNNPGIH
>TEV cleavage site
GPGENLYFQSGSG
>P4
EEEEKKKKEEEEKKKIAQLKQKIQALKQENQQLEEENAALEYG

11	P3h-TEV$_s$-CAD-TEV$_s$-hP4 “PACE”	

>myc
MEQKLISEEDL
>mCherry
VSKGEEDNMAIIKEFMRFKVHMEGSVNGHEFEIEGEGEGRPYEGTQTAKLKVTKGGPLPFAWDILSPQFMYGSKAYVKHPADIPDYLKLSFPEGF
KWERVMNFEDGGVVTVTQDSSLQDGEFIYKVKLRGTNFPSDGPVMQKKTMGWEASSERMYPEDGALKGEIKQRLKLKDGGHYDAEVKTTYK
AKKPVQLPGAYNVNIKLDITSHNEDYTIVEQYERAEGRHSTGGMDELYKSGLRSRAQASNSYAPE
>P3
IQQLEEEIAQLEQKNAALKEKNQALKYEEEEKKKKEEEEKKG
>TEV cleavage site
ENLYFQS
>CAD
ALQKWLQLTHEVEVQYYNIKKQNAEKQLLVAKEGAEKIKKKRNTLFGTFHVAHSSSLDDVDHKILTAKQALSEVTAALRERLHRWQQIEILCGF
QIVNNPGIH
>TEV cleavage site
GPGENLYFQSGSG
>P4
EEEEKKKKEEEEKKKIAQLKQKIQALKQENQQLEEENAALEYG

12	P3h-PPV$_s$-CAD-TEV$_s$-hP4	

>myc
MEQKLISEEDL
>mCherry
VSKGEEDNMAIIKEFMRFKVHMEGSVNGHEFEIEGEGEGRPYEGTQTAKLKVTKGGPLPFAWDILSPQFMYGSKAYVKHPADIPDYLKLSFPEGF
KWERVMNFEDGGVVTVTQDSSLQDGEFIYKVKLRGTNFPSDGPVMQKKTMGWEASSERMYPEDGALKGEIKQRLKLKDGGHYDAEVKTTYK
AKKPVQLPGAYNVNIKLDITSHNEDYTIVEQYERAEGRHSTGGMDELYKSGLRSRAQASNSYAPE
>P3
IQQLEEEIAQLEQKNAALKEKNQALKYEEEEKKKKEEEEKKG
>TEV cleavage site
NVVVHQA
>CAD
ALQKWLQLTHEVEVQYYNIKKQNAEKQLLVAKEGAEKIKKKRNTLFGTFHVAHSSSLDDVDHKILTAKQALSEVTAALRERLHRWQQIEILCGF
QIVNNPGIH
>TEV cleavage site
GPGENLYFQSGSG
>P4
EEEEKKKKEEEEKKKIAQLKQKIQALKQENQQLEEENAALEYG

13	P3h-TEV$_s$-CAD-PPV$_s$-hP4	

>myc
MEQKLISEEDL
>mCherry
VSKGEEDNMAIIKEFMRFKVHMEGSVNGHEFEIEGEGEGRPYEGTQTAKLKVTKGGPLPFAWDILSPQFMYGSKAYVKHPADIPDYLKLSFPEGF
KWERVMNFEDGGVVTVTQDSSLQDGEFIYKVKLRGTNFPSDGPVMQKKTMGWEASSERMYPEDGALKGEIKQRLKLKDGGHYDAEVKTTYK
AKKPVQLPGAYNVNIKLDITSHNEDYTIVEQYERAEGRHSTGGMDELYKSGLRSRAQASNSYAPE
>P3
IQQLEEEIAQLEQKNAALKEKNQALKYEEEEKKKKEEEEKKG
>TEV cleavage site
ENLYFQS
>CAD
ALQKWLQLTHEVEVQYYNIKKQNAEKQLLVAKEGAEKIKKKRNTLFGTFHVAHSSSLDDVDHKILTAKQALSEVTAALRERLHRWQQIEILCGF
QIVNNPGIH
>TEV cleavage site
GPGNVVVHQAGSG
>P4
EEEEKKKKEEEEKKKIAQLKQKIQALKQENQQLEEENAALEYG

14	P3h-PPV$_s$-CAD-PPV$_s$-hP4	

>myc
MEQKLISEEDL
>mCherry
VSKGEEDNMAIIKEFMRFKVHMEGSVNGHEFEIEGEGEGRPYEGTQTAKLKVTKGGPLPFAWDILSPQFMYGSKAYVKHPADIPDYLKLSFPEGF
KWERVMNFEDGGVVTVTQDSSLQDGEFIYKVKLRGTNFPSDGPVMQKKTMGWEASSERMYPEDGALKGEIKQRLKLKDGGHYDAEVKTTYK
AKKPVQLPGAYNVNIKLDITSHNEDYTIVEQYERAEGRHSTGGMDELYKSGLRSRAQASNSYAPE
>P3
IQQLEEEIAQLEQKNAALKEKNQALKYEEEEKKKKEEEEKKG
>TEV cleavage site
NVVVHQA
>CAD
ALQKWLQLTHEVEVQYYNIKKQNAEKQLLVAKEGAEKIKKKRNTLFGTFHVAHSSSLDDVDHKILTAKQALSEVTAALRERLHRWQQIEILCGF
QIVNNPGIH
>TEV cleavage site
GPGNVVVHQAGSG
>P4
EEEEKKKKEEEEKKKIAQLKQKIQALKQENQQLEEENAALEYG
